# Genomes of *Ashbya* Fungi Isolated from Insects Reveal Four Mating-Type Loci, Numerous Translocations, Lack of Transposons, and Distinct Gene Duplications

**DOI:** 10.1534/g3.112.002881

**Published:** 2013-08-01

**Authors:** Fred S. Dietrich, Sylvia Voegeli, Sidney Kuo, Peter Philippsen

**Affiliations:** * Duke Institute of Genome Sciences and Policy, Duke University, Durham, North Carolina 27710; † Department of Molecular Genetics and Microbiology, Duke University, Durham, North Carolina 27710; ‡ Molecular Microbiology, Biozentrum, University of Basel, CH4056 Basel, Switzerland

**Keywords:** fungal ecology, tandem duplications, intron evolution, mating type

## Abstract

The filamentous fungus *Ashbya gossypii* is a cotton pathogen transmitted by insects. It is readily grown and manipulated in the laboratory and is commercially exploited as a natural overproducer of vitamin B2. Our previous genome analysis of *A. gossypii* isolate ATCC10895, collected in Trinidad nearly 100 years ago, revealed extensive synteny with the *Saccharomyces cerevisiae* genome, leading us to use it as a model organism to understand the evolution of filamentous growth. To further develop *Ashbya* as a model system, we have investigated the ecological niche of *A. gossypii* and isolated additional strains and a sibling species, both useful in comparative analysis. We isolated fungi morphologically similar to *A. gossypii* from different plant-feeding insects of the suborder *Heteroptera*, generated a phylogenetic tree based on rDNA-ITS sequences, and performed high coverage short read sequencing with one *A. gossypii* isolate from Florida, a new species, *Ashbya aceri*, isolated in North Carolina, and a genetically marked derivative of ATCC10895 intensively used for functional studies. In contrast to *S. cerevisiae*, all strains carry four not three mating type loci, adding a new puzzle in the evolution of *Ashbya* species. Another surprise was the genome identity of 99.9% between the Florida strain and ATCC10895, isolated in Trinidad. The *A. aceri* and *A. gossypii* genomes show conserved gene orders rearranged by eight translocations, 90% overall sequence identity, and fewer tandem duplications in the *A. aceri* genome. Both species lack transposable elements. Finally, our work identifies plant-feeding insects of the suborder Heteroptera as the most likely natural reservoir of *Ashbya*, and that infection of cotton and other plants may be incidental to the growth of the fungus in its insect host.

The filamentous fungus *A. gossypii* was first isolated from diseased cotton bolls and described as a pathogen of cotton nearly 100 years ago by Ashby (Ashby 1916) and Nowell (Nowell 1915, 1916). They referred to this organism as a member of the “fungus of stigmatomycosis” (Ashby and Nowell 1926) and also realized, as later verified (Pearson 1934; Williams 1934), that the disease of cotton was always associated with specific insects, the cotton stainers, members of the suborder *Heteroptera*. *A. gossypii* played a role in the discovery of biotin (Farries and Bell 1930), and the elucidation of the riboflavin biosynthetic pathway (Bacher *et al.* 2000). The ability of *A. gossypii* to overproduce riboflavin is exploited commercially for the production of this vitamin (Stahmann *et al.* 2000).

The interest in developing *A. gossypii* into a genetically tractable system and to conduct genomic sequencing began when synteny with *S. cerevisiae* was discovered (Altmann-Jöhl and Philippsen 1996) and when it was shown that, unlike other filamentous fungi, homologous recombination was the rule, not the exception (Steiner *et al.* 1995). The genome sequence of the *A. gossypii* strain (ATCC10895), the source for the development of riboflavin overproducers and for biological investigations (Philippsen *et al.* 2005; Wendland and Walther 2005), revealed greater than 90% synteny of the annotated protein-coding genes with the gene set of *S. cerevisiae* (Dietrich *et al.* 2004). The *A. gossypii* genome sequence has been used in numerous comparative genome studies (Fisk *et al.* 2006; Gordon *et al.* 2009; Seret *et al.* 2009; Souciet *et al.* 2009) and for experimental studies aimed at understanding the evolution of budding yeast and filamentous fungus life styles starting from the same ancestral set of genes (Wendland and Philippsen 2001; Wendland 2003; Philippsen *et al.* 2005; Gladfelter *et al.* 2006; Knechtle *et al.* 2006; Schmitz *et al.* 2006; Koehli *et al.* 2008b; DeMay *et al.* 2009; Kaufmann and Philippsen 2009; Grava and Philippsen 2010; Grunler *et al.* 2010; Lang *et al.* 2010; Nair *et al.* 2010; Finlayson *et al.* 2011; Jorde *et al.* 2011; Gibeaux *et al.* 2013). The genome sequence is also of commercial interest, being used to identify ways to increase riboflavin production in *A. gossypii* (Kato and Park 2012).

Until the recent genomic sequencing of *Eremothecium cymbalariae*, *A. gossypii* was the only sequenced fungal genome of a species related to budding yeast growing in a strictly filamentous mode with multinucleated and multibranching hyphae (Schmitz and Philippsen 2011; Wendland and Walther 2011). To find out whether this combination, budding yeast-like genome and growth as multinucleated hyphae, is rare, we aimed to analyze additional strains and species of *Ashbya* isolated from nature. For example, by comparing *Ashbya* genomes, we wanted to determine whether important tandem gene duplications and gene losses described for *A. gossypii* ATCC10895 (Koehli *et al.* 2008a; Kaufmann and Philippsen 2009) are specific properties of that strain or are conserved in other *Ashbya* isolates. It was also important to analyze the mating type loci in novel isolates, as the ATCC10895 genome only carries *MATa* copies and lacks *MAT*α information. Finally, we expected to define the environmental niche in which these organisms are found.

## Materials and Methods

### Strains, media, and polymerase chain reaction (PCR) primers

More than 30 new wild *A. gossypii* strains were isolated from large milkweed bugs (*Oncopeltus fasciatus*) feeding on oleander (*Nerium oleander*) or on common milkweed (*Asclepias syriaca*). Six *A. aceri* strains were obtained from the eastern boxelder bug (*Boisea trivittata*) collected from boxelder trees (*Acer negundo*) and maple trees (*Acer* sp.). In addition, three *Ashbya* strains were isolated from the western boxelder bug (*Boisea rubrolineata*) feeding on maple trees (*Acer* sp.) and seven *Ashbya* strains from the red-shouldered bug (*Jadera hematoloma*) feeding on golden raintrees (*Koelreuteria paniculata*). Fungal isolation was performed by crushing the juvenile or adult insects on yeast extract peptone dextrose (Sherman *et al.* 1987) or *Ashbya* full media (Altmann-Jöhl and Philippsen 1996) with ampicillin (100 µg/mL) and tetracycline (100 µg/mL) added to limit bacterial growth. A range of filamentous fungi and yeasts were growing on the plates, but in most cases it was possible to identify colonies resembling those of *A. gossypii* strain ATCC10895. Mycelium from these colonies was restreaked to pure culture and stored at −80°. DNA isolations were performed using a standard yeast protocol (Sherman *et al.* 1987) except that the fungi were collected by filtration instead of centrifugation. Internal transcribed sequence (ITS) sequences were generated using the ITS1 and ITS4 primers (ITS1: 5′TCCGTAGGTGAACCTTGCGG3′; ITS4: 5′TCCTCCGCTTATTGATATGC3′). *Ashbya* spp. were the only fungi consistently isolated from these insects. The *A. gossypii* strain Agleu2Δthr4Δ was obtained by targeted deletion of the *LEU2* and *THR4* genes of *A. gossypii* strain ATCC10895 and screening for subsequent excision of the selectable marker. Thus, the deletions are unmarked and contain no foreign DNA (Altmann-Jöhl and Philippsen 1996).

### Sequencing strategy

Genomic sequencing was performed using genomic DNA prepared by standard methods (Sherman *et al.* 1987) using the short read Solexa sequencing technology from Illumina (www.illumina.com) (Bentley 2006). Sequences were generated at GATC (www.gatc-biotech.com) resulting in 36 million 36 base reads for each of the two *A. gossypii* genomes, and at Duke University in the group of Kevin Shiana resulting in 15 million reads for the *Ashbya aceri* genome. An additional 29 million pairs of 58 base long Illumina Solexa mate pair sequences were generated at GATC for insect isolate 1 and 31 million pairs of 58 base long mate pair sequences for *A. aceri*. The 36 base pair sequence reads were assembled with the use of a standard heuristic hash-based algorithm coded in C and compiled under gcc 4.2 (http://gcc.gnu.org/). To summarize in brief, the sequence and quality score were compressed to 2 bits for the sequence, 2 bits for the quality score. Sequences that were identical or differed only at low quality bases were combined. Reads that overlapped by 35 of 36 bases were then combined to create initial contigs. Branch points were identified as divergence of multiple high quality bases. The initial contigs were then combined by sequentially joining of contigs of decreasing overlap down to 20 bases while blocking extension at identified branch points.

Once contig assembly was completed, the depth of coverage was calculated for each contig and a scaffold was created. It should be noted that this algorithm was successful in assembling the single read 36 base pair reads into less than 800 contigs for each strain primarily because *A. gossypii* has no transposable elements, has a GC content of close to 50%, and has very few repetitive sequences. Contigs and scaffolds were then assembled by alignment to the original *A. gossypii* sequence (Dietrich *et al.* 2004) using FASTA (Lipman and Pearson 1985), Basic Local Alignment Search Tool, *i.e.*, BLAST (Altschul *et al.* 1997), and LAGAN (Brudno *et al.* 2003). When ambiguities occurred, the original chromatograms and pairing information from that project was investigated. Most valuable were the 80- to 100-kb bacterial artificial chromosome (BAC) end sequences from the earlier project in scaffolding the 36 base pair derived contigs. Multiple ambiguities were present in the assemblies based solely on the 36 base reads, which were addressed by using the pairing information from the 58 base mate pair reads. Additional assemblies were carried out using maq (Li *et al.* 2008) and velvet (Zerbino and Birney 2008); contigs from these assemblies were aligned to the assembly described previously and each discrepancy was individually investigated. Further confirmation of the sequence was obtained by aligning sequence reads back to the completed sequence using BWA (Li and Durbin 2009) and SAMtools (Li *et al.* 2009) and regions of discrepancy were investigated. Investigation of ambiguities in the assemblies was carried out using a set of scripts to carry out exhaustive local alignments. Phylogenetic analysis was carried out using clustalx (Thompson *et al.* 1994).

### Annotation of the assembled genomes

Annotation was performed with standard tools, including BLAST (Altschul *et al.* 1997), FASTA (Lipman and Pearson 1985), C, Perl, BioPerl (Stajich *et al.* 2002), EMBOSS (Rice *et al.* 2000), weblogo (Crooks *et al.* 2004), TBl2asn, and Sequin (http://www.ncbi.nlm.nih.gov/Sequin). All gene names were maintained from the original annotation, with the exception that ACR186W is the syntenic ortholog of YJR080C (*AIM24*) and ACR185W is the syntenic ortholog of YJR082C (*EAF6*). The names were erroneously reversed in the original annotation. New open reading frames (ORFs) were named by the upstream ORF adding an A or B after the systematic name, *e.g.*, ADL139C-A. All ORFs from the *A. gossypii* insect isolate 1 from Florida were named like the ORFs from strain ATCC10895 but adding an F before the systematic name, *e.g.*, FABL001 for the first gene on the left arm of chromosome 2. All ORFs from the *A aceri* insect isolate 38 were also named following the *A. gossypii* nomenclature of the reference strain ATCC10895 irrespective of the translocations but adding an “Aaceri” prior to the systematic name.

## Results

### Fungi associated with insects belonging to the subfamily *Heteroptera*

**Figure 1:**
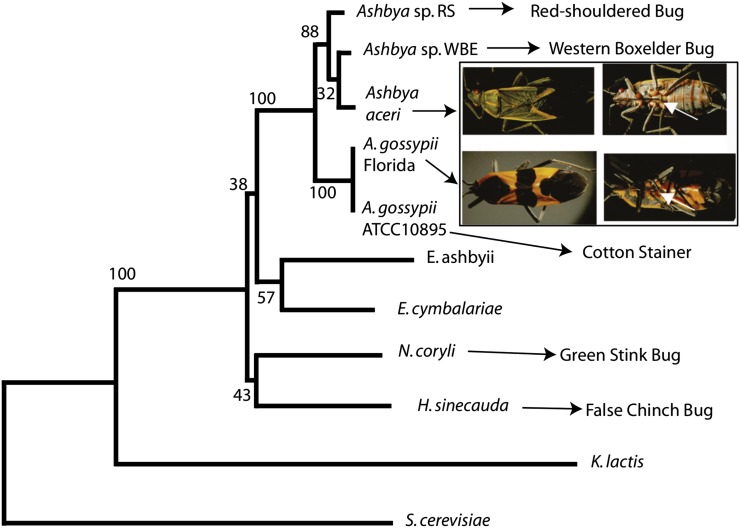
Fungi most closely related to *A. gossypii*. Neighbor joining phylogenetic tree of the known and newly isolated *Nematosporaceae* based on ITS sequences, along with *Kluyveromyces lactis*, and with *S. cerevisiae* as an outgroup. *Ashbya* sp. RS isolated from red-shouldered bugs and *Ashbya* sp. western boxelder (WBE) isolated from western boxelder bugs are as-yet uncharacterized beyond ITS sequencing. *A. aceri* is a fungus isolated from an eastern box elder bug shown in the insert. Its complete genome sequence was determined during this work. *A. gossypii* Florida isolate was isolated from a large milkweed bug shown in the insert. Its complete genome sequence also was determined during this work. The white arrows in the insect images indicate the probosci through which these insects feed, and through which the fungus is transmitted between the plant and the insect. *A. gossypii* has been described before to be spread by the cotton stainer (Ashby and Nowell 1926; Frazer 1944). *A. gossypii* ATCC10895 refers to the reference strain for all *Ashbya* species the genome of which was resequenced for the comparative analyses presented in this study This *A. gossypii* reference strain from the American Type Culture Collection was isolated from diseased cotton (Ashby 1916) and most likely originated from the US Agricultural Research Service strain collection (NRRL Y-1056), where it was obtained from William J. Robbins, who reported obtaining it from the Centraalbureau Voor Schimmelcultures (CBS) (Robbins and Schmidt 1939), where *A. gossypii* had been deposited by S. F. Ashby in 1926 (CBS 109.26), and possibly the same strain was deposited by Alexandre Guilliermond in 1928 (CBS 117.28). *Holleya sinecada* has been reported to be spread by the False Chinch bug (Burgess and McKenzie 1991), and *Nematospora coryli* by the Green stink bug (Clarke and Wilde 1970). Specific insect species have not been associated with *Eremothecium cymbalariae* or *Eremothecium ashbyii*. It has been suggested that all of the fungi of the family *Nematosporaceae* are spread by heteropterous insects (Batra 1973). GenBank accession numbers for the ITS sequences of the other *Nematosporaceae* are U09326.1 for *Nematospora coryli*, FJ422506.1 for *Holleya sinecauda*, AY046219.1 for *Eremothecium cymbalariae*, AB478315.1 for *Eremothecium ashbyii*, AJ229069.1 for the yeast *Kluyveromyces lactis*, and NC_001144 for *Saccharomyces cerevisiae*. The available ITS sequence data cannot well resolve the structure of the tree at the base of the *Nematosporaceae* clade.

**Figure 2:**
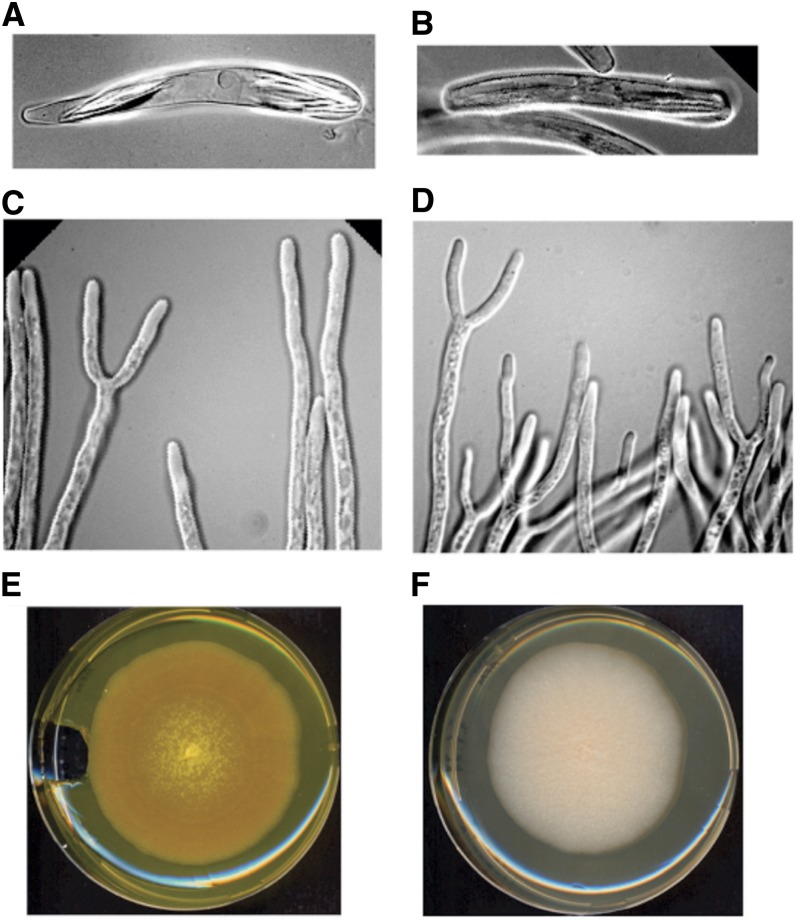
Growth of *A. gossypii* insect isolate 1 strain and *A. aceri* (insect isolate 38). (A) Ascus from insect isolate 1. (B) Ascus from insect isolate 38. (C) Hyphal tip branching of insect isolate 1. (D) Hyphal tip branching of insect isolate 38. (E) Hyphal mat formed by insect isolate 1. (F) Hyphal mat formed by insect isolate 38. No aerial mycelial growth was observed.

We hypothesized that Ashbya-like fungi may be associated with insects related to the cotton stainer, which belongs to the *Heteroptera* subfamily. Indeed, *Ashbya* strains could be isolated from adults and juveniles, but not eggs, from large milkweed bugs found feeding on oleander in Florida, the U.S. Virgin Islands, and North Carolina and on common milkweed in North Carolina and Virginia (Figure 1). All fungal isolates from these insect species grew as multinucleated lateral branching and tip-splitting hyphae, produced needle-shaped spores, and appeared to be riboflavin overproducers based on the characteristic diffusible yellow coloration of colonies typical for *A. gossypii* (Figure 2). All tested isolates had identical ITS1 and ITS2 sequences to that of *A. gossypii* ATCC 10895, the reference strain. The new *A. gossypii* strain (insect isolate 1), whose genome sequence is described below, was isolated from a large milkweed bug collected on oleander near Miami, Florida, in August 2005.

After the isolation of new strains of *A. gossypii* from large milkweed bugs, additional *Heteroptera* species were examined. Eastern boxelder bugs were collected in North Carolina, Wisconsin, and New York and tested for the presence of this fungus. In each case, including in over-wintering bugs from New York, a fungus could be isolated from these insects that by appearances is quite similar to *A. gossypii*, but colonies grown for a week on yeast extract peptone dextrose or *Ashbya* full media plates have a white-to-cream color and no sign of the yellow color that is ubiquitous among the *A. gossypii* isolates (Figure 2F). On the basis of the ITS sequence, these organisms appear closely related to *A. gossypii* but most likely represent a new *Ashbya* species (Figure 1). The *Ashbya* strain associated with an eastern boxelder bug, collected in North Carolina from a boxelder tree in August 2007 (insect isolate 38), was selected for genome sequencing because CHEF gel analysis of its karyotype had revealed different chromosome sizes but a similar number of chromosomes to that of *A. gossypii* (Supporting Information, Figure S1). This isolate is here named *Ashbya aceri* after the genus name *Acer* of the boxelder and maple trees on which the insects harboring this fungus feed.

Ashbya-like fungi could also be isolated from western boxelder bugs feeding on maple trees in California and New Mexico and from red-shouldered bugs feeding on golden rain tree in North Carolina. ITS sequences from these fungi are similar but not identical to *A. gossypii* ITS sequences. For one isolate each, a genome analysis is in progress. All ITS sequences determined during this screening study are different from the known ITS sequences of the other *Nematosporaceae* species shown in the phylogenetic tree of Figure 1.

### Re-annotation of *A. gossypii* ATCC10895 based on sequencing of Agleu2Δthr4Δ

To perform a reliable comparative analysis with the newly sequenced genomes, we at first had to correct sequence errors and fill gaps in the genome of the *A. gossypii* reference strain ATCC10895. The original sequences were generated using dideoxy shotgun sequencing and clone walking of plasmid and BAC clones using paired-end information for assembly purposes, and sequencing of PCR products to close gaps. The overall fourfold sequence coverage of the 9 MB genome gave an average sequence accuracy of 99.8% (Dietrich *et al.* 2004). To establish a highly accurate sequence we decided not to resequence the ATCC10895 genome but the genome of the host strain for functional analyses, derived from the ATCC10895 strain by targeted gene deletions of *AgLEU2* and *AgTHR4* followed by excision of the selection markers (Altmann-Jöhl and Philippsen 1996). Using the high throughput Illumina sequencing technology (Bentley 2006) 17,336,954 sequences of 36 bases in length were generated. The sequence assembly of this 35-fold coverage short read sequence data were consistent with the gene order previously reported, and the analysis suggests that the finished sequences (with the *AgLEU2* and *AgTHR4* sequences added) represent the entire genome of *A. gossypii* strain ATCC10895.

Combining the short read sequence data with the original *A. gossypii* genome sequence resulted in identification and correction of more than 10,000 sequence errors. These included 8301 substitutions, 668 one- to five-base deletions, and 369 one- to five-base insertions, where most of the insertion/deletion errors were of a single base (Figure S2). The sequence across the three previous gaps has been completed, although in one case, that of a poly-C stretch in the upstream noncoding region of AFL160C, the sequence quality is low. The sequence has been completed to the telomere terminal repeats for all 14 chromosome ends, reaching the terminal 24-bp telomeric repeat, TGAGAGACCCATACACCACACCGC. A complete re-annotation of the genome, taking advantage of both the sequence corrections and the genomic sequences of additional species published since the initial release of the *A. gossypii* genome has resulted in an updated set of the seven *A. gossypii* chromosomes (GenBank accession numbers AE016814 through AE016820). For the mitochondrial genome, no errors were detected and its annotation remained unchanged (AE016821).

The reannotation added 31 protein-coding genes, most notably a fourth copy of MATa that was identified at the right subtelomeric region of chromosome VI discussed below. In addition, 3 noncoding RNA genes, 15 introns, and 1 transfer RNA (tRNA) gene (Table 1, Table 2, and Table 3) were added. A total of 15 protein coding genes, 3 tRNA genes, and 1 noncoding RNA gene were deemed incorrect and removed. The coding capacity of the reference strain now encompasses 4776 proteins, 221 tRNAs, 83 small RNAs, and 35 copies of rDNA. The reannotation also corrected the amino acid sequence of proteins at 1165 positions, and it increased or decreased the length of 152 open reading frames, primarily as a result of changes at their 5′ end. There are two defective genes: AFR753C contains multiple stop codons and is a syntenic homolog of *S. cerevisiae* YNL246W (VPS75); the other is an apparently defective copy of a leucine tRNA. Reannotation also identified eight genes that are apparently translated across frameshifts. These genes include homologs of four genes translated across frameshifts in *S. cerevisiae* (ADL016C − *EST3*, ACR130W − *ABP140*, AGL265W − *OAZ1*, and ABR148CA − YJR112W-A), and four genes additional genes (ACR287W − *ATS1*, ADR251W − *CIN4*, AGR057C *IOC2*, and AFR597W). The AFR597W gene appears to be a case of −1 frameshifting; the other seven are +1 frameshifting. AFR597W has no homolog in *S. cerevisiae* but is similar to *S. kluyveri* SAKL0H03652g.

**Table 1:** Genes added to the annotation of *A. gossypii* strain ATCC10895

Location Added:	Feature	Position	Strand	A.g. ORF Name	S.c.1° Homolog	S.c.2° Homolog	S.c.1° Homolog Common Name	S.c.2° Homolog Common Name
Chr1	CDS	189767..189973	+	AAL088W-A	NOHBY121			
Chr2	CDS	2047..2991	+	ABL211W	NOHBY219			
Chr2	ncRNA	286717..286792	−	AgSNR86	SNR86			
Chr2	ncRNA	404973..405057	+	AgSNR87	SNR87			
Chr2	5′UTR	458552..458694	−	5′ UTR intron				
Chr2	CDS	862752..863330	+	ABR250W	NOHBY220			
Chr2	CDS	867475..867642	−	ABR251C	NOHBY221			
Chr3	CDS	222051..222212	+	ACL074W-A	NOHBY335			
Chr3	CDS	471357..471584	+	ACR063W-A	YIL102C-A			
Chr4	CDS	10088..10672	−	ADL397C-B	NOHBY454			
Chr4	CDS	10088..10672	−	ADL397C-B	NOHBY454			
Chr4	CDS	11914..12576	−	ADL397C-A	NOHBY453			
Chr4	CDS	11914..12576	−	ADL397C-A	NOHBY453			
Chr4	CDS	83599..84255, 84309..84314	−	ADL356C-A	NOHBY452			
Chr4	5′UTR	153033..153181	+	5′ UTR intron				
Chr4	CDS	240411..241580	+	ADL265W-A	YOR099W		KTR1	
Chr4	CDS	430981..431586	−	ADL148C	NOHBY450			
Chr4	CDS	434768..435013	+	ADL147W-A	YGR236C		SPG1	
Chr4	5′UTR	472058..472560	−	5′ UTR intron				
Chr4	CDS	593768..594280	−	ADL052C-A	NOHBY451			
Chr4	CDS	712125..712700	+	ADR004W	NOHBY422			
Chr4	5′UTR	877761..878116	+	5′ UTR intron				
Chr4	CDS	921189..921761	+	ADR121W-A	NOHBY455			
Chr5	ncRNA	22986..23002	−	AgSNR50	SNR50			
Chr5	CDS	98045..98974	+	AEL289W-A	YKR005C			
Chr5	5′UTR	318815..318910	+	5′ UTR intron				
Chr5	CDS	471962..472540	−	AEL083C-A	NOHBY534			
Chr5	CDS	563288..565171	+	AEL037W	NOHBY503			
Chr5	5′UTR	733654..733809	+	5′ UTR intron				
Chr5	5′UTR	800547..800684	−	5′ UTR intron				
Chr5	CDS	939696..941048	−	AER160C-A	NOHBY536			
Chr5	CDS	1017648..1018565	+	AER204W-A	NOHBY535			
Chr5	5′UTR	1105232..1105307	+	5′ UTR intron				
Chr5	CDS	1132015..1133691	−	AER270C-A	NOHBY533			
Chr5	5′UTR	1325900..1326076	−	5′ UTR intron				
Chr6	CDS	412760..413410	−	AFL013C	NOHBY671			
Chr6	5′UTR	790825..790986	+	5′ UTR intron				
Chr6	5′UTR	791737..791787	+	5′ UTR intron				
Chr6	tRNA	1251500..1251538, 1251548..1251583	+	tRNA-Tyr	tRNA-Tyr			
Chr6	CDS	1251695..1252657	+	AFR451W-A	YDL209C		CWC2	
Chr6	5′UTR	1300412..1300621	−	5′ UTR intron				
Chr6	CDS	1802735..1803337	+	AFR742W	NOHBY668			
Chr7	5′UTR	181129..181250	−	5′ UTR intron				
Chr7	CDS	573775..574320	+	AGL073W-C	YMR073C		IRC21	
Chr7	CDS	574575..575699	−	AGL073C-A	YKL032C	YMR072W	IXR1	ABF2
Chr7	CDS	575980..577428	−	AGL073C-B	NOHBY747			
Chr7	CDS	619742..619921,619973..620005	−	AGL048C	YPR010C-A			
Chr7	CDS	673624..674040	+	AGL024W-A	NOHBY748			
Chr7	5′UTR	945704..945829	−	5′ UTR intron				
Chr7	CDS	1291412..1292176	−	AGR294C-A	NOHBY749			
Chr7	5′UTR	1426879..1427068	+	5′ UTR intron				

ORF, open reading frame; CDS, coding sequence; ncRNA, noncoding RNA; UTR, untranslated region; tRNA, transfer RNA.

**Table 2:** Genes removed from the annotation of *A. gossypii* strain ATCC10895

Location Deleted:	Feature	Position	Strand	A.g. ORF Name	S.c.1° Homolog	S.c.2° Homolog	S.c.1° Homolog Common Name	S.c.2° Homolog Common Name
Chr2	snRNA	286517..286773	+	AgSNR81	SNR81			
Chr3	CDS	564351..564956	+	ACR121W	NOHBY323			
Chr3	CDS	711557..712282	−	ACR208C	NOHBY331			
Chr4	CDS	114447..114788	−	ADL337C-A	YBR108WX			
Chr4	CDS	430731..431204	+	ADL148W	NOHBY411			
Chr4	tRNA	683817..683888	−	tRNA-Sec	tRNA-Sec			
Chr4	CDS	712052..712354	−	ADR004C	NOHBY422			
Chr4	CDS	937410..938060	+	ADR129W	NOHBY428			
Chr4	CDS	1380467..1380700	+	ADR376W	NOHBY446			
Chr5	tRNA	102468..102503,102525..102562	−	tRNA-Gln	tRNA-Gln			
Chr5	tRNA	103053..103090,103112..103147	+	tRNA-Gln	tRNA-Gln			
Chr5	CDS	564134..565534	−	AEL037C	NOHBY503			
Chr6	CDS	411639..412373	+	AFL013W	NOHBY602			
Chr6	CDS	1120279..1121118	+	AFR379W	NOHBY639			
Chr6	CDS	1799446..1800129	−	AFR742C	NOHBY668			
Chr7	CDS	616255..616749	+	AGL048W	NOHBY705			
Chr7	CDS	913067..913483	+	AGR094W	NOHBY739			
Chr7	CDS	944867..945574	+	AGR109W	NOHBY740			
Chr7	CDS	1308521..1309006	−	AGR307C	NOHBY745			

ORF, open reading frame; snRNA, small nuclear RNA; CDS, coding sequence; ncRNA, noncoding RNA; tRNA, transfer RNA.

**Table 3:** Genes annotations modified for *A. gossypii* strain ATCC10895

Location Change of Status	Feature	Position	Strand	A.g. ORF Name	S.c.1° Homolog	S.c.2° Homolog	S.c.1° Homolog Common Name	S.c.2° Homolog Common Name
Former								
Chr1	CDS	548298..548819	+	AAR114W	NOHBY113			
Chr1	CDS	637960..638250	−	AAR163C	NOHBY116			
Chr2	CDS	670947..671588	−	ABR143C	YGR272C			
Chr3	CDS	670703..672574	+	ACR185W	YJR080C		FMP26	
Chr3	CDS	670809..671105	−	ACR186W	YJR082C		EAF6	
Chr4	CDS	136074..138218	−	ADL318C	YBR098W		MMS4	
Chr4	CDS	652348..653358	+	ADL027W	YAL018C			
Chr4	tRNA	681277..681348	−	tRNA-Asn	tRNA-Asn			
Chr4	tRNA	683817..683888	−	tRNA-Sec	tRNA-Sec			
Chr4	CDS	1266115..1267167	+	ADR318W	NOHBY438			
Chr4	tRNA	1280568..1280617, 1280766..1280803	−	pseudo tRNA-Leu	tRNA-OTHER			
Chr5	CDS	566187..566612	+	AEL036W	NOHBY502			
Chr5	CDS	756469..757149	+	AER066W	NOHBY513			
Chr7	CDS	104690..104863	+	AGL322W	YBL039W-A		YBL039W-B	
Current								
Chr1	CDS	548273..548794	+	AAR114W	YLR224W			
Chr1	CDS	637934..638224	−	AAR163C	YPL187W	YGL089C	MFΑ1	MFΑ2
Chr2	CDS	670766..671506	−	ABR143C	YGR271C-A		EFG1	
Chr3	CDS	670815..671111	+	ACR186W	YJR080C		AIM24	
Chr3	CDS	671405..672580	+	ACR185W	YJR082C		EAF6	
Chr4	CDS	136197..138341	−	ADL318C	YBR098W		MMS4	
Chr4	CDS	652405..653415	+	ADL027W	YOL048C	YAL018C		
Chr4	tRNA	671190..671262	+	tRNA-Thr	tRNA-Thr			
Chr4	tRNA	683864..683935	−	tRNA-Arg	tRNA-Arg			
Chr4	CDS	1266092..1267144	+	ADR318W	YLR224W			
Chr4	tRNA	1280543..1280592,1280741..1280778	−	tRNA-Leu	tRNA-Leu			
Chr5	CDS	565382..565807	+	AEL036W	YFL017C	YOL024W		
Chr5	CDS	755657..756337	+	AER066W	YDL069C		CBS1	
Chr7	CDS	104819..104992	+	AGL322W	YBL039W-B			

ORF, open reading frame; CDS, coding sequence; tRNA, transfer RNA.

All but 181,456 sequence reads were used in the genome assembly. More than 90% of these remaining reads are low quality or are apparently bacterial and *S. cerevisiae* contamination. The only unused sequence reads that assembled into contigs using velvet (Zerbino and Birney 2008) were variants of the canonical 24 bp *A. gossypii* terminal telomeric sequence, arising from a result of a high rate of sequence variation.

The overall 35-fold short read sequence coverage appeared to be very close to randomly distributed across these genomes. There was one gap in both the short read sequence data of strain ATCC10895 and insect isolate 1, described below, and these gaps were at the same location, in a polyC region in the non-coding sequence adjacent to AFL160C, the *A. gossypii* homolog of *GAL4* located on chromosome VI. Efforts to PCR across this region have been unsuccessful, strongly suggesting that this gap results from a technical difficulty.

An additional deviation from randomness is found in the Agleu2Δthr4Δ strain sequence, there are 80 short regions of 1 to 83 bases where sequence coverage is less than eightfold coverage. All but two of these are short stretches that are either more than 85% GC or less that 15% GC. All of these regions were checked by visual inspection.

Based on synteny and protein similarity, the *A. gossypii* nuclear genome appears to encode 4776 protein coding genes, 4300 (90%) of which have syntenic homologs in *S. cerevisiae*, and another 171 (3.6%) of protein coding genes have nonsyntenic homologs, leaving 270 (5.7%) of the protein coding genes in *A. gossypii* with no homolog in Baker’s yeast (NOHBY). A comparison with the more closely related *Kluyveromyces lactis* sequence (Dujon *et al.* 2004) and other sequenced fungal genomes identifies 146 of the 270 NOHBY genes (54%) as having a syntenic homolog in at least one species, and 24 of the 260 NOHBY genes (9.2%) with at least one nonsyntenic homolog. Thus, currently only 90 protein coding genes identified in *A. gossypii*, or less than 2% of the predicted proteins, have no apparent homolog in other fungi.

### Sequencing *A. gossypii* insect isolate 1

**Figure 3:**
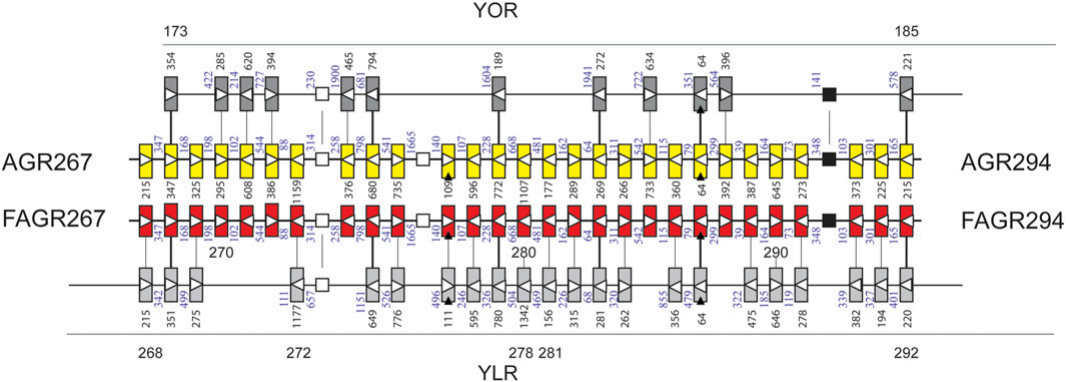
Synteny between orthologous chromosomal regions of *A. gossypii* and *S. cerevisiae*. The yellow and red rectangles represent ORFs 267−294 of the right arm of chromosome 7 of *A. gossypii* ATCC10895 and insect isolate 1, respectively. The dark gray and light gray rectangles represent *S. cerevisiae* ORFs from the right arm of chromosome XV (above) and the left arm of chromosome XII (below), which are syntenic to the *A. gossypii* ORFs. Open triangles show transcription directions and filled arrow heads mark ORFs with intron. Open squares are tRNA genes and closed squares small nuclear RNA genes. The gene order is conserved between the two *A. gossypii* strains and also the lengths of the ORFs (number of codons) and the sizes of the inter-ORF regions (number of base pairs). The synteny with *S. cerevisiae* is divided between two chromosomal regions. At the time of the *S. cerevisiae* genome duplication both regions showed complete synteny to the *A. gossypii* gene order. During evolution many of the duplicated genes lost one copy seen as ORF-free regions in this synteny map. The synteny map also reveals six cases (five ORFs and one tRNA gene) where both copies of the duplication are retained. To distinguish these duplications from tandem duplications the term twin genes was coined (Dietrich *et al.* 2004).

We also performed short-read sequencing of insect isolate 1. A total of 17,134,963 sequences of 36 bases in length assembled into eight contigs using as template the updated genome of the *A. gossypii* reference strain. The initial assembly of the genome sequence using only the single read Illumina sequence reads allowed assembly of most of the genome but could not resolve the sequence of small repetitive regions, particularly the subtelomeric sequence. An additional 58,091,226 sequences were generated using the Illumina Mate Pair strategy (www.illumina.com) consisting of 18,674,012 pairs of sequence reads with insert lengths averaging 1.6 kb in length where both ends can be aligned to the genome. The pairing data provided sufficient information to complete the assembly across the repetitive regions of the genome, providing the organizational information that was obtained by BAC and plasmid end pair sequence data for the genomic sequence of strain ATC10895. Both genomes have the same gene order. The genome sequence of insect isolate 1 is 99.9% identical to that of ATCC10895, and thus shares the high level of synteny with the budding yeast genome previously reported (Figure 3). The deposited sequence reveals only 15,337 single-nucleotide polymorphisms (SNPs), 424 single-base insertion/deletions differences (indels), and 952 indels of more than one base relative to ATCC10895.

**Figure 4:**
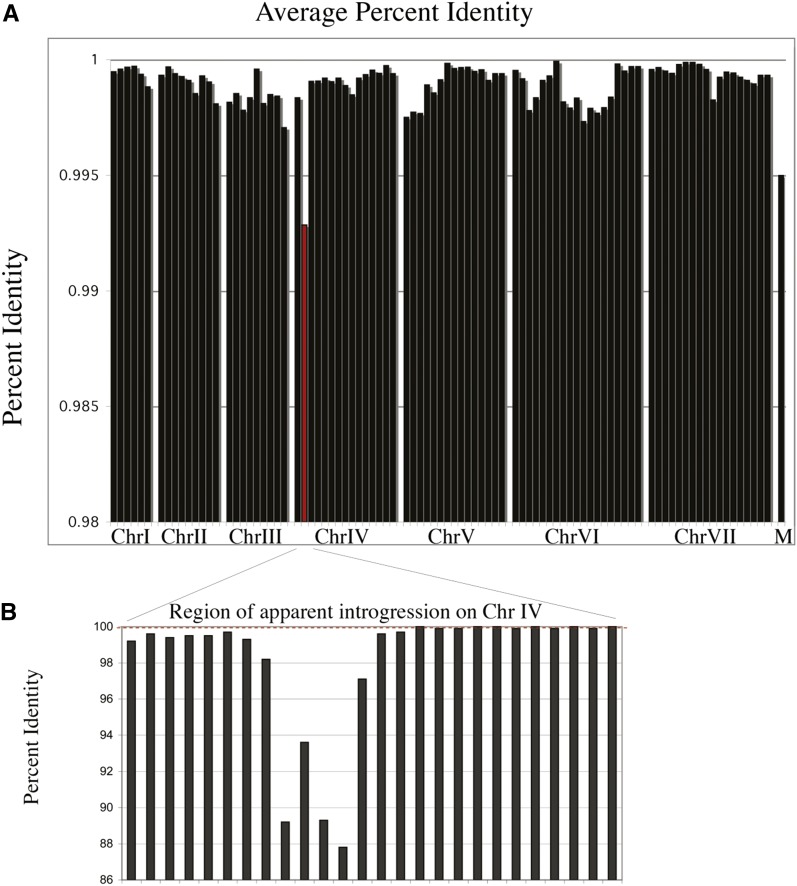
Blocks of sequence conservation of up to 450 genes between ATCC10895 and insect isolate 1. (A) Distribution of sequence identity across the genomes was averaged over 100-kb intervals reveals that some regions are more similar, and some more diverged. On chromosome V the region from approximately 501,000 to 1,270,000, spanning 410 protein coding genes is 99.96% identical between these strains. On chromosome VI the region from approximately 700,000 to 1,478,000, spanning 434 protein coding genes, is 99.80% identical between these strains. The mitochondrial genome labeled “M” is more diverged than the nuclear genome. The telomeric regions of chromosomes V, VI, and VII show more sequence divergence, particularly rearrangements in repetitive elements, than the genome overall and are not shown in this figure. The nuclear genomes are on average 99.9% identical, excluding the telomeric regions. (B) A syntenic region of 5450 bases of significantly lower homology, approximately 92% identity, between *A. gossypii* strains ATCC10895 and insect isolate 1 is found on chromosome 4 (red bar in A), with boundaries from 179,139 to 184,589 bases in ATCC10895. Percent identity was averaged over windows of 1 kb. Of the 439 SNPs in the introgression region, 139 are in inter-ORF regions, which have an average identity of 92.6%. The remaining 300 SNPs, 186 synonymous and 97 nonsynonymous, fall in the four open reading frames of this region, ADL294C, ADL295W, ADL296C, and ADL297W, which have an average identity of 91.6%. Interestingly, one of the genes, ADL296C, encodes the enzyme GTP cyclohydrolase, the first step in riboflavin biosynthesis. Although the introgressed regions are 92% identical to each other, they are both approximately equally diverged from *A. aceri* at only 78% identity each, suggesting the source of the introgression is not *A. aceri*, but another *Ashbya* species more closely related to *A. gossypii*.

A total of 63% of the SNPs are purine/purine or pyrimidine/pyrimidine transitions (see Table S1). These polymorphisms are distributed somewhat unevenly across the genome, as seen in Figure 4. One 5-kb region on chromosome IV containing four protein-coding genes, ADL294 to ADL297, is only 92% identical between the two strains. This region, which interestingly encodes a key enzyme for riboflavin synthesis, accounts for nearly 5% of the polymorphisms seen in the nontelomeric regions between these two strains and appears to be an introgression event in which one of these strains has obtained this sequence from a closely related species. This introgression is similar to those reported in *S. cerevisiae* and *S. paradoxus* (Liti *et al.* 2006). The subtelomeric regions, particularly the chromosome VI right end, contribute nearly half of all SNPs, and more than half of all indel differences seen between these strains. One of the few genes showing multiple polymorphisms between these strains is ABR072C. In contrast to *S. cerevisiae* where the homolog is a single copy of the cell wall mannoprotein, *CWP1*, both *A. gossypii* strains sequenced have four copies of this gene, at a syntenic location. Two of these genes contain internal tandem repeats. In ABR027C, these internal repeats are differentially arranged in the two *A. gossypii* sequences.

### MAT gene in novel *Ashbya* isolates

**Figure 5:**
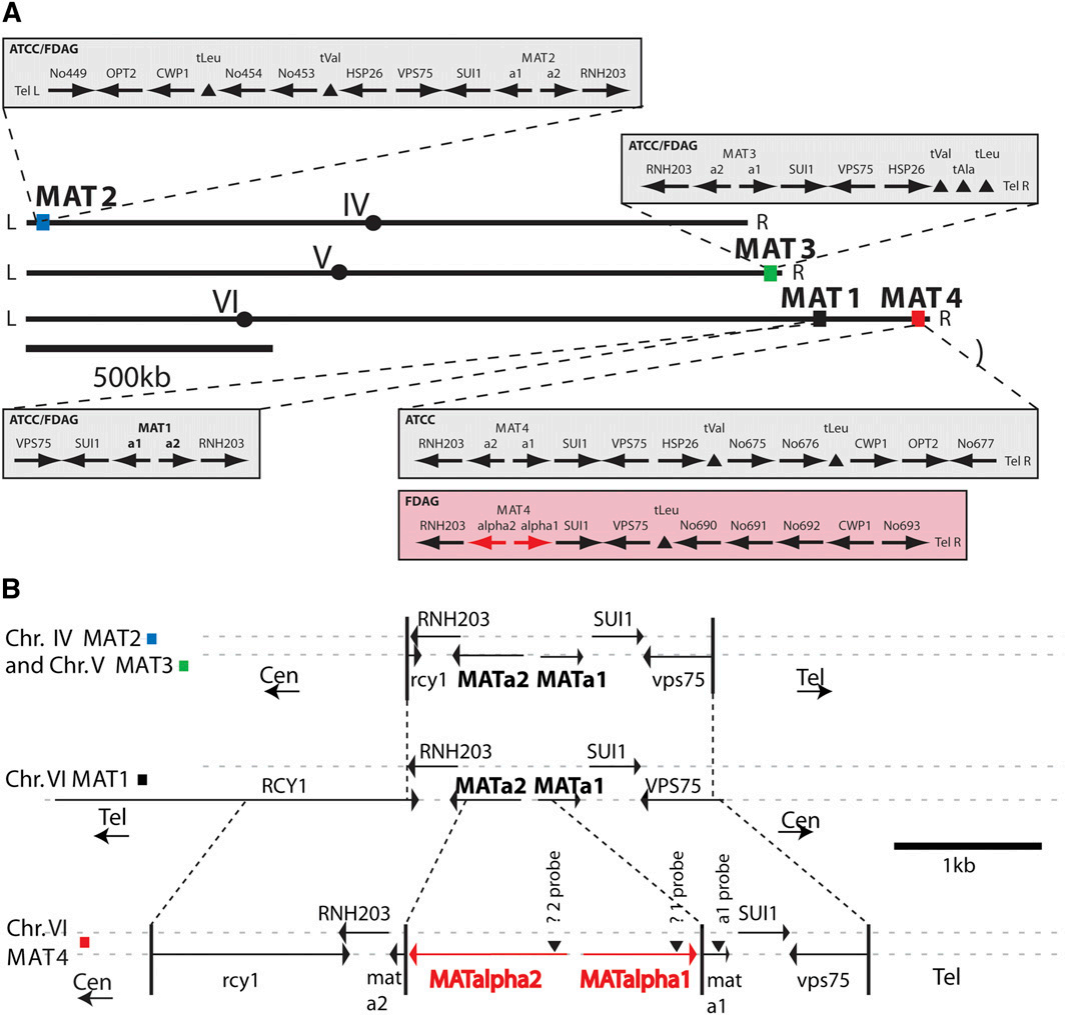
*A. gossypii* mating type regions of ATCC10895 and the insect isolate 1 strain. (A) Overall organization of the four mating type loci on chromosomes IV, V, and VI. The three chromosomes are shown in the orientation as annotated. Circles mark the centromere locations; colored squares mark the locations of the mating type loci *MAT1* to *MAT4*. The enlarged sections show the genetic map of these regions in both strains. No differences were found except for the *MAT4* locus at the right telomere of chromosome 6 that carries *α1/α2* information in the Florida isolate and *a1*/*a2* information in ATCC10895. Interestingly, the order of genes distal to MAT1 and MAT4 is identical in ATCC10895. It is therefore very likely that the *MAT4* locus of ATCC10895 originally carried *α1/α2* genes, like the Florida isolate, which were replaced with *a1*/*a2* genes by a gene conversion event with the left telomere of chromosome 4 initiated by a break in the homology region around *RNH203* proximal to *MAT4*. Table S2 presents the nomenclature of genes associated with the four MAT loci. (B) Fine structure of the four MAT loci of the Florida isolate and ATCC10895 before the gene conversion at MAT4. All gene names refer to the *S. cerevisiae* homologs, except for *a2*, which is a homolog of the *K. lactis MATa2* gene (Astrom *et al.* 2000). The telomeric loci on chromosomes IV and V are flanked by partial copies of the *RCY1* and *VPS75* genes, marked in lower case. Vertical bars and dotted lines indicate the junctions of homology at the mating type loci, the centromeric and telomeric ends being marked by “Cen” and “Tel.” Only the nontelomeric MAT1 locus is flanked by intact *RCY1* and *VPS75* genes, suggesting that this locus on chromosome VI is the active mating type locus, with ATCC10895 and the Florida isolate 1 being MATa. The orientation shown is opposite of that in part A. The chromosome VI telomeric MAT4 locus in the Florida strain carries *MATα2* and *MATα1* genes inserted into remnants of *MATa2* and *MATa1* genes, indicated in lower case. The locus is somewhat larger, containing more sequence from the still truncated *RCY1* and *VPS75* genes. MATα specific sequences are shown in red. The sequence arrangements at the MAT loci were confirmed by DNA hybridizations using synthetic oligonucleotides with homology to the positions indicated by arrow heads (data not shown).

Interestingly, the genome of the insect isolate carries two additional genes not found in the reference strain ATCC10895. These genes are orthologs of *MATα1* (YCR040W) and *MATα2* (YCR039C) genes of *S. cerevisiae* and map at the right subtelomeric region of chromosome VI, which harbors in the reference strain the originally overlooked fourth *MATa* copy (Figure 5). In both *A. gossypii* and *S. cerevisiae* this pair of genes are divergently transcribed. This strain also carries three copies of the *MATa* genes, one at the presumptive active locus on the right arm of chromosome VI and the other two at subtelomeric regions of chromosomes IV and V like in the reference strain. Five other wild isolates of *A. gossypii* also encode both *MATa* and *MATα* sequences, based on PCR assays (data not shown). The lack of *MATα* sequences in the genome of ATCC10895 and the 100% sequence identity distal to the MAT loci in the sub-telomeric regions of chromosome IV and chromosome VI suggests that the MATα genes together with the distal portion of chromosome VI were lost by a gene conversion event as indicated in Figure 5A. This event possibly occurred during the lengthy passaging of ATCC10895 in the laboratory.

**Figure 6:**
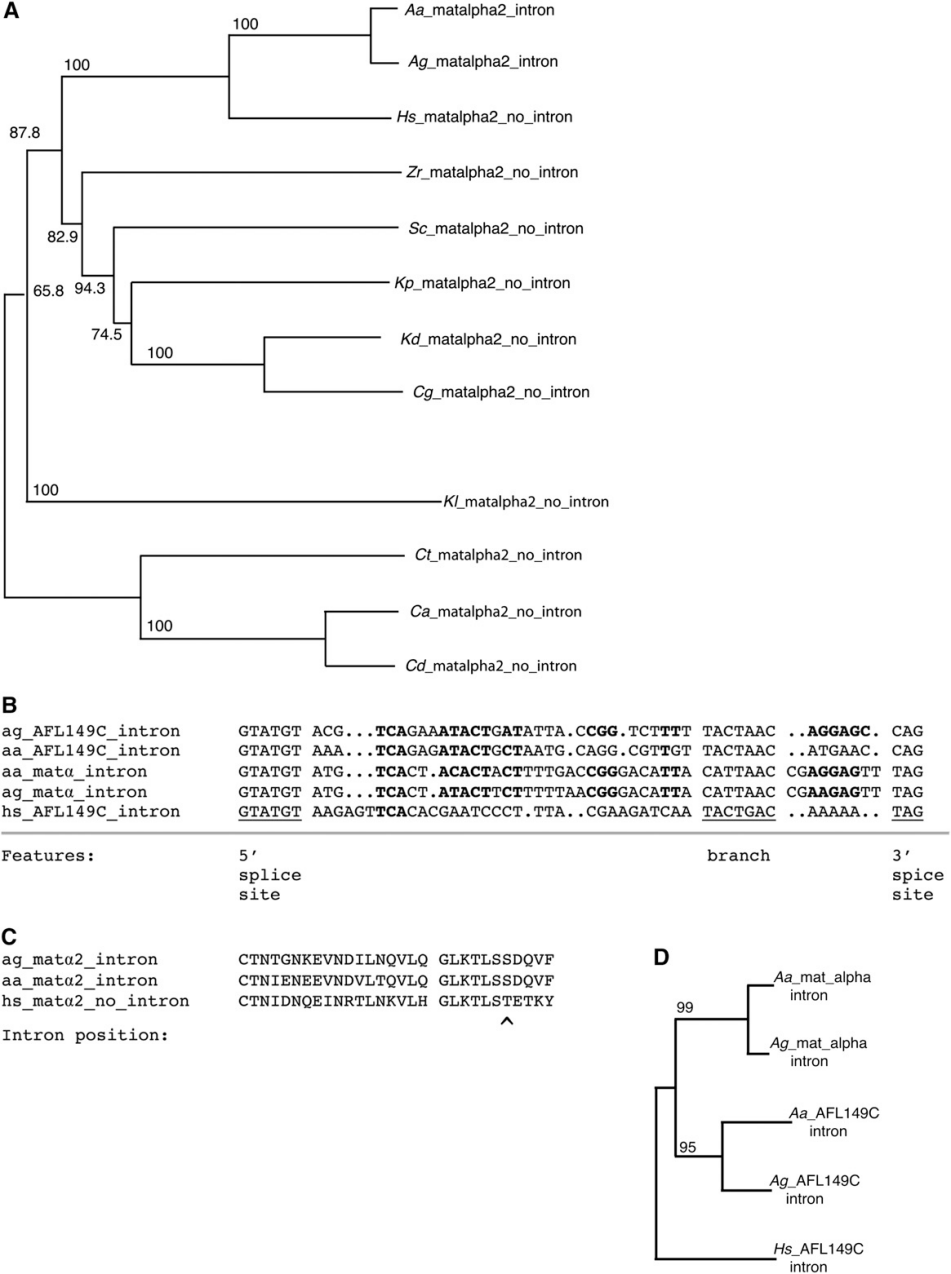
An unusual intron in *Ashbya MATα2*. (A) A neighbor joining phylogenetic tree of the MATα2 protein. Numbers refer to bootstrap values. Species are *A. aceri* (Aa), *A. gossypii* (Ag), *H. sinecauda* (Hs), *S. cerevisiae* (Sc), *Kluyveromyces* (*Vanderwaltozyma*) *polyspora* (Kp) (Scannell *et al.* 2007), *Kluyveromyces delphensis* (Kd) (Wong *et al.* 2003), *Candida glabrata*, (Cg) (Dujon *et al.* 2004), *Zygosaccharomyces rouxi* (Zr) (Souciet *et al.* 2009), *Kluyveromyces lactis* (Kl) (Dujon *et al.* 2004), *Candida thermotolerans* (Ct) (Souciet *et al.* 2009), *Candida albicans* (Ca) (Hull and Johnson 1999), and *Candida dubliniensis* (Cd). (B) Alignment of the introns of *A. gossypii* gene AFL149C with the homologous introns from *H. sinecauda* and *A. aceri*, and the introns from the *MATα2* gene of *A. gossypii* and *A. aceri*. An intron at this position is found in no other *MATα2* genes currently available in GenBank. The 5′ splice site, branch point, and 3′ splice site are marked. Conserved sequence within the intron is marked in bold. (C) A partial alignment of the *Ashbya* and *H. sinecauda MATα2* proteins is shown with the position of the intron marked. The intron is outside the conserved homeobox domain. The alignment suggests no sequences have been gained or lost at the site of this intron. (D) A phylogenetic tree of the intron sequences of MATα2 and AFL149C.

Unlike *S. cerevisiae* and other available sequences, the *AgMATα2* gene contains an intron, as does the *MATα2* genes in three *Candida* species (Figure 6A), although at a different position. The intron sequences in *MATα2* of *A. gossypii* and *A. aceri* (see below) are shown in Figure 6B. The sequence of this intron has weak sequence similarity to the intron sequence of AFL149C (Figure 6, B and D). The *MATα2* and AFL149C coding regions have no obvious DNA or protein sequence homology at the splice sites (data not shown).

### Introns in *A. gossypii*

**Figure 7:**
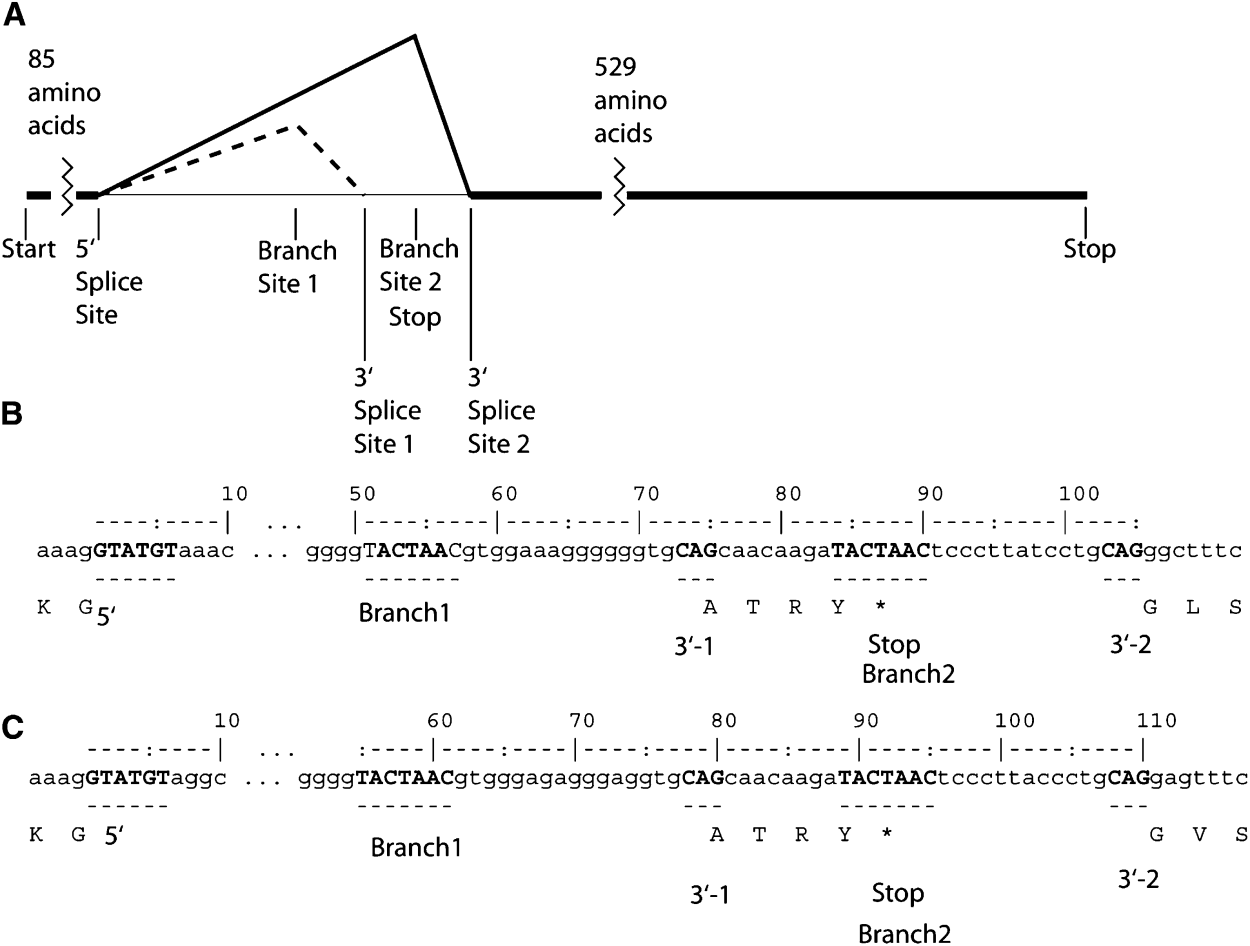
Unique case of an intron with two 3′ splice sites in *Ashbya*. (A) The intron in *A. gossypii* ADR221C is shown to scale with the two branch points and two 3′ splice sites. (B) The sequence of the *A. gossypii* ADR221C intron, total size 105 nucleotides for the longer form. The 5′ Splice site, two branch points, and two 3′ splice sites are shown in upper case, with the translation below. (C) *A. aceri* ADR221C intron, with a total size 110 nucleotides for the longer form.

A total of 263 protein-coding genes in both *A. gossypii* genomes contain a single intron, seven have two introns, and 49 tRNA genes contain an intron. An additional 15 introns are located in the 5′ UTR of protein coding genes. The intron splice consensus sequence for protein coding genes is very similar to that of *S. cerevisiae* as shown in Figure S3, although the average length of introns in *A. gossypii* (107 bases) is less than half of the average length of introns in *S. cerevisiae* (244 bases). Only one gene, ADR221C, contains an intron that in both *Ashbya* species has two of the preferred branch point sequences and two 3′ splice sites. Both of these possible 3′ splice sites are in-frame, although there is a stop codon between them, so that one of the possible splices will result in an mRNA with an in-frame premature stop codon, whereas the splicing of the longer form of the intron will bypass the stop codon (Figure 7). An unusually large intron is found at the same position in the coding region and in the same reading frame in *Candida albicans* (420nt), *Kluyveromyces thermotolerens* (978nt), *Zygosaccharomyces rouxi* (752 nt), *K. lactis* (342), and *K. polysporus* (1 gene, 660nt). In each of these cases the intron appears to have only a single 3′ splice site. In *S. cerevisiae* and *Candida glabrata* the duplicate copies resulting from the genome duplication have been retained, though the intron has been lost from both copies. In *S. cerevisiae*, the two orthologs are *SKI7* and *HBS1*. The *SKI7* gene has been shown to play a role in degrading mRNA containing premature stop codons (van Hoof *et al.* 2000) and the structure of the intron in the *A. gossypii* homolog of *SKI7* suggests a possible novel mechanism of feedback regulation in this gene.

### Genome sequence of *Ashbya aceri* isolated from a boxelder bug

We have carried out short read genomic sequencing of the *A. aceri* strain insect isolate 38 and assembled its genome from 36 million single 36 base sequence reads and an additional 30 million mate pairs of 58 base long Illumina data. Problems arose at GC rich sequences, at break points of translocations, at telomeres, and particularly at the homologs of *A. gossypii* AFL095W and AFL092C. These genes are a tandem inverted duplicate pair homologs of *S. cerevisiae FLO5* with nearly 8 kb of internally repetitive sequence between them in both *Ashbya* species. Although multiple genes containing internal inverted repeats are found in *S. cerevisiae* (Verstrepen *et al.* 2005), none are in this convergent tandem orientation that potentially allows for diversity to be generated by inversions between repetitive sequences. Most of these problems could be solved by visual inspections. The DNA sequence of *A. aceri* is 90% identical to that of *A. gossypii* strain ATCC10895 and contains eight reciprocal translocations not including those at telomeres (Table 4). The genome has three *MATa* loci and one *MATalpha* locus at positions identical to *MAT* loci of the *A. gossypii* insect isolate 1 (Figure 5). Protein identity ranges from 40 to 100% with an average of 89% identity compared with *A. gossypii*. The lowest identity was found for a protein encoded by AFR028W, a gene present at syntenic positions in many yeasts, but with unknown function in *S. cerevisiae*. Other proteins with low identity are encoded by NOHBYs, genes with no homolog in Baker’s yeast, but present in *Ashbya* and in some cases other related fungi. The gene order is highly conserved between these species, other than at the mentioned translocation breakpoints.

**Table 4:** Locations of translocations between *A. aceri* and *A. gossypii*

Chromosome in *A. aceri*	Chromosome in *A. gossypii*	Gene to Left of Break Point		Gene to Right of Break Point	Chromosome in *A. gossypii*	Translocation Type
AaChr7	AgChr7	AGR242C	—	ADR160W	AgChr4	Reciprocal
AaChr4	AgChr4	ADR159C	—	AGR243W	AgChr7	*A.g*. Ancestral
AaChr3	AgChr6	AFR280W	—	ACL074W	AgChr3	Reciprocal
AaChr6	AgChr6	AFR279C	—	ACL075C	AgChr3	*A.a*. Ancestral
AaChr2	AgChr4	ADR357C	—	AGR346C	AgChr7	Reciprocal
AaChr4	AgChr7	AGR345C	—	ADR358W	AgChr4	*A.g*. Ancestral
AaChr1	AgChr1	AAR190W	—	AEL287C	AgChr5	Reciprocal
AaChr5	AgChr1	AAR191C	—	AEL286C	AgChr5	*A.g*. Ancestral
AaChr7	AgChr4	ADR165C	—	AER303W	AgChr5	Adjacent
AaChr5	AgChr5	AER302C	—	ADR166W	AgChr4	Reciprocal
AaChr7	AgChr7	AGR242C	—	ADR160W	AgChr4	Translocations
AaChr4	AgChr4	ADR159C	—	AGR243W	AgChr7	Unclear ancestry
AaChr2	AgChr2	ABR182W	—	AFR451W-A	AgChr6	Adjacent
AaChr3	AgChr2	ABR183W	—	AFR451C	AgChr6	Reciprocal
AaChr2	AgChr6	AFR466C	—	ABR186W	AgChr2	Translocations
AaChr3	AgChr6	AFR467W	—	ABR184C	AgChr2	Unclear ancestry
AaChr6	AgChr4	ADL268C	—	AFL185W	AgChr6	Adjacent
AaChr7	AgChr4	ADL267W	—	AFL186W	AgChr6	Reciprocal
AaChr4	AgChr5	AER443W	—	ADL265W-A	AgChr4	Translocations
AaChr7	AgChr5	AER442W	—	ADL265W	AgChr4	*A.g*. Ancestral
AaChr7	AgChr3	ACL203C	—	AGL351W	AgChr7	Telomeric
AaChr6	AgChr7	AGL352W	—	ADL395C	AgChr4	Gene exchange
AaChr3	AgChr4	ADL397C	—	AFR747W	AgChr6	Unclear ancestry
AaChr2	AgChr2	ABR208W	—	ADR329W	AgChr4	Three way
AaChr3	AgChr3	ACR239C	—	ABR209W	AgChr2	Translocation
AaChr5	AgChr4	ADR328W	—	ACR240W	AgChr3	*A.a*. Ancestral

*A. aceri* chromosomes are numbered and oriented based on the conserved centromeric regions. No translocations are present at the centromeres. The first column lists the *A*. aceri chromosome. *A. gossypii* protein coding genes listed are adjacent to the translocation. Four of the translocations are reciprocal translocations and the other five are either a pair of translocations at near-by genes, or are three-way transloctions. In most cases it is possible to identify if the *A. aceri* or *A. gossypii* gene order is ancestral by comparison with related species *E. cymbalariae* (Wendland and Walther 2011) and *K. lactis* (Dujon *et al.* 2004).

Only a few genes differences were noted between the *A. aceri* and the *A. gossypii* genomes. For example, *A. aceri* lacks two tandem duplications, one triplication and one quadruplication found in both sequenced *A. gossypii* genomes (see Table 5). *A. aceri* carries only one syntenic homolog of the *S. cerevisiae CDC123* gene involved in nutritional control of the cell cycle and only one syntenic homolog of the *S. cerevisiae RAI1* gene involved in decapping of mRNAs (Bieganowski *et al.* 2004; Jiao *et al.* 2010).

**Table 5:** Tandem duplications of protein coding genes in *A. gossypii* strains

*A. gossypii* Gene	Identity*^a^*	*S. cerevisiae* Homolog 1	*S. cerevisia* Homolog 2*^b^*	*A. gossypii**^c^*	*A. aceri**^c^*
AAL179W	47%	YJL079C(PRY1)	YKR013W(PRY2)	2	2
AAL178W					
ABL189W	38%	YDL237W		2	2
ABL188W					
ABR025C	26–46%	YKL096W(CWP1)		4	4
ABR026C					
ABR027C					
ABR028C					
ACR272C	58%	YKL096W(CWP1)		2	2
ACR273W					
AFL095W	56%	YHR211W(FLO5)		2	2
AFL092C					
ABR182W	73%	YPR165W(RHO1)		2	2
ABR183W					
ABR246W	65–84%	YIR035C	YIR036C	**4**	**0**
ABR247W					
ABR248W					
ABR249W					
ACL202W	81–94%	YMR238W(DFG5)		**3**	**1**
ACL201W					
ACL200W					
ACR098C	45%	YPL129W(TAF14)	YOR213C(SAS5)	2	2
ACR099C					
ACR143W	36%	YPL154C(PEP4)		2	2
ACR144W					
ADL156C	66%	YOL119C(MCH4)		2	2
ADL155C					
ADR081C	82%	YLR215C(CDC123)		**2**	**1**
ADR082C					
ADR336C	60%	YNR055C(HOL1)		2	2
ADR337C					
ADR403C	25–27%	YAL051W(OAF1)	YOR363C(PIP2)	3	3
ADR404C					
ADR405C					
AER452C	74–80%	YJR107W		3	3
AER453C					
AER454C					
AFR262C	87%	YGL246C(RAI1)		**2**	**1**
AFR263C					
AGL352W	59%	YMR307W(GAS1)		2	2
AGL351W					
AGL326W	29%	YJL172W(CPS1)		2	2
AGL325W					
AGR038C	72%	YDR046C(BAP3)	YBR068C(BAP2)	2	2
AGR039C					
AGR188W	<30%	YDR227W(SIR4)		2	2
AGR189W					
AGR405C	83%	YCL057W(PRD1)		2	2
AGR406C					

a Identity between tandem gene pair in *A. gossypii*.

b Tandem copies in *S. cerevisiae*, or gene duplicate pairs.

c Number of tandem genes at respective location; bold indicates differences between *A. gossypii* and *A. asceri*.

These two genes are tandemly duplicated in *A. gossypii*. Furthermore, one syntenic and one telomeric copy of the *S. cerevisiae DFG5* gene, encoding a mannosidase essential for cell wall biosynthesis (Kitagaki *et al.* 2002), are present in *A. aceri*. Interestingly, in *A. gossypii* the telomeric copy has amplified to a tandem triplication. Finally, *A. aceri* and *A. gossypii* each carry one syntenic homolog of the *S. cerevisiae* tandem gene duplication YIR035C/036C encoding putative benzyl reductases which could be involved in detoxification reactions (Maruyama *et al.* 2002). *A. gossypii* additionally carries a tandem quadruplication of this putative reductase gene, absent in *A. aceri*, near the right telomere of chromosome 2.

The three sequenced *Ashbya* genomes carry a gene, (AGL178W), with homology to the reverse transcriptase of the *S. cerevisiae* TY3 elements, though these species lack transposable elements. There is no evidence of introgression between *A. aceri* and *A. gossypii*, with the largest region of >95% sequence identity being the rDNA. We are confident that this organism represents a new species of the genus *Ashbya* and here give the following description:

*Ashbya aceri* Nov. sp. ; *Ashbya* Guilliermond
Isolated from *Boisea trivittata* found on *Acer negundo*. Hyphal mat color white to cream. Hyphae fail to invade agar. Some aerial mycelia. Hyphae, with lateral and tip branching. Yeast cells not observed. Asci arise from vegetative mycelium. Ascospores needle-shaped, typically 8 per ascus.
Etymology: From genus *Acer*, maple and boxelder trees, M. Latin *aceri*, from *Acer*.
The genus *Ashbya* was defined by Alexandre Guilliermond (Guilliermond 1928).

## Discussion

It is yet to be shown if the relationship between the “fungi of stigmatomycosis” and the insects in which they are found represents a symbiosis or is a commensal relationship. It is possible that these fungi provide nutrients that allow these insects to live on the plants on which they are found, in a manner analogous to that seen in insects that harbor symbiotic bacteria, such as *Buchnera* (Lai and Baumann 1992). This finding is consistent with the observation that these fungi typically are found in the mouth parts of these insects (Frazer 1944; Foster and Daugherty 1969).

The overproduction of riboflavin in *Ashbya* strains found in insects living on milkweed and oleander plants that produce toxic alkaloids (Everist 1981; Lewis and Elvin-Lewis 1986). The lack of such overproduction in strains isolated from insects found on the non-toxic boxelder and maple trees may be explained by the hypothesis that overproduction of riboflavin allows the insects to live on alkaloid-producing toxic plants using a mechanism of detoxification of alkaloids using flavin cofactor (Miranda *et al.* 1991; Cashman *et al.* 1996; Sehlmeyer *et al.* 2010; Langel and Ober 2011).

Although there has so far been only one member of the genus *Ashbya*, it is quite possible that this is largely due to a lack of sampling. The known species of the *Nematosporaceae* are all associated with the plant feeding bugs of the suborder *Heteroptera*, including the fungi *Holleya sinecauda* and *N. coryli* that have been associated with the false chinch bug, *Nysius ericae* (Burgess *et al.* 1983; Burgess and Weegar 1986) and green stink bugs, *Acrosternum hilare* (Clarke and Wilde 1970), respectively (Figure 1). There are conservatively estimated to be 35,000 species of *Heteroptera* (Slater 1982), though some such as the assassin bug (family Reduviidae) feed on other insects, not on plants, and when tested did not appear to carry a specific fungus (data not shown).

Comparative analysis of completely sequenced genomes can be used to reveal the frequency and distribution of SNPs, and the conservation of gene arrangements, *e.g.*, presence of introns or overlapping transcripts, functional gains by tandem duplications or modifications of pathways by specific gene losses. The distribution of polymorphisms across the two *A. gossypii* genomes as shown in Figure 4 suggests that there has been reassortment of genetic material in the wild so that some portions of these two genomes are more closely related than other parts. Although this could result either from a sexual cycle or a parasexual cycle, the presence of both the *MATa* and *MATalpha* loci in wild isolates of *Ashbya* is similar to what is found in other Hemiascomycetes, and suggests that *A. gossypii* likely has a sexual cycle.

More than 200 of the 270 intron containing protein coding genes in *A. gossypii* have an intron containing homolog in *S. cerevisiae*, with intron loss in the *S. cerevisiae* lineage being the most likely explanation for the remaining cases. It has long been speculated that introns in *S. cerevisiae* may have been lost by a mechanism involving reverse transcription and gene conversion (Fink 1987), and an example in *Cryptococcus neoformans* where this appears to have occurred has recently been described (Stajich and Dietrich 2006). The intron found in the *MATα2* gene of *A. gossypii* insect isolate1 and *A. aceri* appears to be a case of intron loss in multiple lineages, though the possibility of intron gain cannot be ruled out (Figure 6D).

After resequencing and reannotating of strain ATCC10895, there are no cases of protein coding ORFs currently annotated that overlap at the 5′ end except for *RNH203* adjacent to the *MAT* loci (Figure 5). The original annotation reported an overlap at the 5′ end for 15 pairs of ORFs. In 8 cases the 5′ ends of transcripts could be determined leading to reannotations of the start codon downstream of the originally annotated start codons (data not shown). The GenBank files of these ORFs were updated, and the ORF pairs no longer overlapped. The remaining cases were validated by multiple alignments with sequences from other available genomes and it is clear that these were also cases with an incorrectly annotated start codons. The homology began at the second or even third or fourth ATG. There are, however, 26 pairs of convergently transcribed ORFs that overlap at their 3′ ends. These are not hypothetical genes, but genes conserved at least among the *Hemiascomycetes*. Examination of the sequence in these regions of overlap, and homology with orthologs of other species strongly suggests that these overlaps are real. Some had already been described before the release of the complete *A. gossypii* sequence (Brachat *et al.* 2003). The overlaps are typically quite short, less than 10 nucleotides. The longest overlap in *A. gossypii* is between AEL311W and AEL310C, overlapping by 172 nucleotides. In *A. aceri* these two ORFs overlap by 166 nucleotides.

The tandemly duplicated genes in each *Ashbya* species are shown in Table 5. Of the 21 sets of duplicated genes in *A. gossypii*, 17 are also tandemly duplicated in *A. aceri* suggesting that these are clade specific and not strain or species specific duplications. Interestingly, the unique functional divergence of the tandemly duplicated *A. gossypii RHO1* genes (ABR182W and ABR183W) is conserved in *A. aceri*. In these fungi the GTPases Rho1a and Rho1b are functionally diverged, with a change of the usually conserved tyrosine to histidine in the switch I region of Rho1a, introducing a novel specificity for a GTPase activating protein and also influencing the localization of Rho1a (Koehli *et al.* 2008a). Another interesting case is the duplication of the *SIR4* gene (AGR188W and AGR189W) encoding an important protein for gene silencing. In *S. cerevisiae*, a heterodimer encoded by the *SIR3* and *SIR4* gene plays a key role in gene silencing (Rusche *et al.* 2003), but the two *Ashbya* species analyzed here lacks a specific *SIR3* gene, which in the *S. cerevisiae* lineage evolved from an *ORC1* duplication. Another unique feature is the conservation of a tandem triplication of a lipase gene (AER452C to AER454C) and a quadruplication of *CWP1*, a cell wall gene, (ABR025C to ABR028C) in both *Ashbya* species. For each of these genes, only a single syntenic copy is present in the *S. cerevisiae* genome. The genome of the related fungus *E. cymbalariae* carries 8 of the 21 tandem gene duplications found in *A. gossypii* including *RHO1* and *SIR4* but lacks among others the mentioned lipase gene triplication and the *CWP1* quadruplication. It also lacks nontandemly repeated Ashbya genes (Table S3), for example the five *MNT3* homologs encoding mannosyl transfereases and other telomere located gene amplifications. Furthermore, compared with *Ashbya* genomes the genome of *E. cymbalariae* has a much lower GC-content (40% *vs.* 52%), carries one additional chromosome, shows over 200 genome rearrangements, and the average protein identity is only 60% compared to *A. gossypii*, which is similar to the average protein identity between the distantly related yeasts *S. cerevisiae* and *K. lactis* (Wendland and Walther 2011).

A group of gene losses was recently associated with the ability of *A. gossypii* hyphae to substantially accelerate their elongation speed (Kaufmann and Philippsen 2009). These genes are also absent in *A. aceri*. The orthologous genes in *S. cerevisiae* encode an endochitinase (*CTS1*), an endoglucanase (*EGT2*) and a cell wall protein (*SCW11*) important for cell separation. The absence of these genes is likely essential for acceleration of hyphal growth, as cell separation does not occur at the occasionally forming septa in *Ashbya*. The multinucleated apical hyphal compartments increase in length over time concomitantly increasing the cytoplasmic space for assembling secretory vesicles. The higher the rate of secretory vesicle production and transport to the hyphal tips, the faster the tips grow, a principle apparently conserved in *Ashbya* fungi.

*Ashbya* genomics can contribute significantly to our understanding of the ecological importance of this genus. First, insects free of fungi can be colonized at will with different *Ashbya* species or, most importantly, with designed mutants allowing examination of the role of genes when the organism is grown in its native environment. For example, large milkweed bugs grown in containers at the Carolina Biological Supply Company (www.carolina.com) lack *A. gossypii*, though these insects could take up and maintain *A. gossypii* when fed on sunflower seeds that had been injected with this fungus (F. D. unpublished data). And second, unlike *S. cerevisiae* that has been isolated from numerous environments, *A. gossypii* specifically, and the fungi of stigmatomycosis in general, have only been isolated from the mouthparts of specific insects from the suborder *Heteroptera*. Not only does the large number of *Heteroptera* species provide the opportunity to isolate additional strains and species of these fungi for comparative analysis, but it also provides the opportunity to investigate how the specific environment in which these fungi live has shaped their genomes.

## Supplementary Material

Supporting Information
