# Supplementary material for: Genomes of Ashbya Fungi Isolated from Insects Reveal Four Mating-Type Loci, Numerous Translocations, Lack of Transposons, and Distinct Gene Duplications
Source: G3 (Bethesda). 2013 Aug 1;3(8):1225–39. doi: 10.1534/g3.112.002881 (PMC3737163; doi:10.1534/g3.112.002881)
Supplement: Supporting Information [file supp_3_8_1225__index.html]

Genomes of Ashbya Fungi Isolated from Insects Reveal Four Mating-Type Loci, Numerous Translocations, Lack of Transposons, and Distinct Gene Duplications — Supporting Information 

# Genomes of *Ashbya* Fungi Isolated from Insects Reveal Four Mating-Type Loci, Numerous Translocations, Lack of Transposons, and Distinct Gene Duplications

## Supporting Information for Dietrich *et al.*, 2013

**Files in this Data Supplement:**

- Supporting Information - Figures S1-S3 and Tables S1-S3 (PDF, 3 MB)
- Figure S1 - Pulsed field gel of A. gossypii strain ATCC10895 (lanes 7,8) and of insect isolate 1 (lanes 1,2), A. aceri (lanes 5, 6), and an A. gossypii isolate from a milk weed bug living on oleander in Vera Beach Florida (lanes 3,4) (PDF, 1.5 MB)
- Figure S2 - Sequence corrections (PDF, 249 KB)
- Figure S3 - Intron Splice sites from *A. gossypii* and *S. cereivisiae* (PDF, 819 KB)
- Table S1 - SNPs and INDELs seen between strains ATCC10895 and insect isolate 1 (PDF, 75 KB)
- Table S2 - Changes to names of mating type and mating type associated *A. gossypii* genes (PDF, 76 KB)
- Table S3 - Names of repeated *A. gossypii* genes with a single *S. cerevisiae* homolog (PDF, 350 KB)
