# Supplementary material for: Genomes of Ashbya Fungi Isolated from Insects Reveal Four Mating-Type Loci, Numerous Translocations, Lack of Transposons, and Distinct Gene Duplications
Source: G3 (Bethesda). 2013 Aug 1;3(8):1225–39. doi: 10.1534/g3.112.002881 (PMC3737163; doi:10.1534/g3.112.002881)
Supplement: Supporting Information [file supp_g3.112.002881_002881SI.pdf]

G3·Genes | Genomes | Genetics

Genomes of *Ashbya* fungi isolated from insects reveal four mating-type loci, numerous translocations, lack of transposons, and distinct gene duplications

Fred S. Dietrich[*], Sylvia Voegeli[§], Sidney Kuo[*], Peter Philippsen[§]

Affiliations

[*] Duke Institute of Genome Sciences and Policy, and Department of Molecular Genetics and Microbiology, Duke University, Durham, North Carolina 27710

[§] Molecular Microbiology, Biozentrum, University of Basel, CH4056 Basel, Switzerland

DOI: 10.1534/g3.112.002881

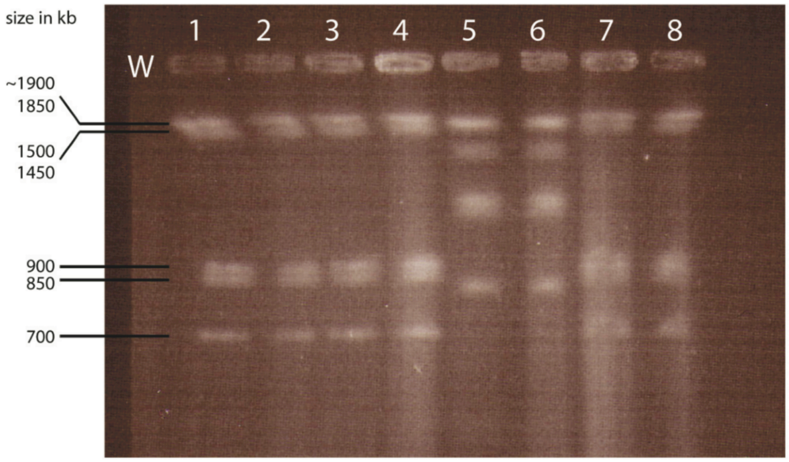

Figure S1 Pulsed field gel of *A. gossypii* strain ATCC10895 (lanes 7,8) and of insect isolate 1 (lanes 1,2), *A. aceri* (lanes 5, 6), and an *A. gossypii* isolate from a milk weed bug living on oleander in Vera Beach Florida (lanes 3,4) Chromosomal size heterogeneity is apparent between these species. W indicates the position of the loading wells. Sizes based on sizes of the *A. gossypii* strain ATCC10895 chromosomes.

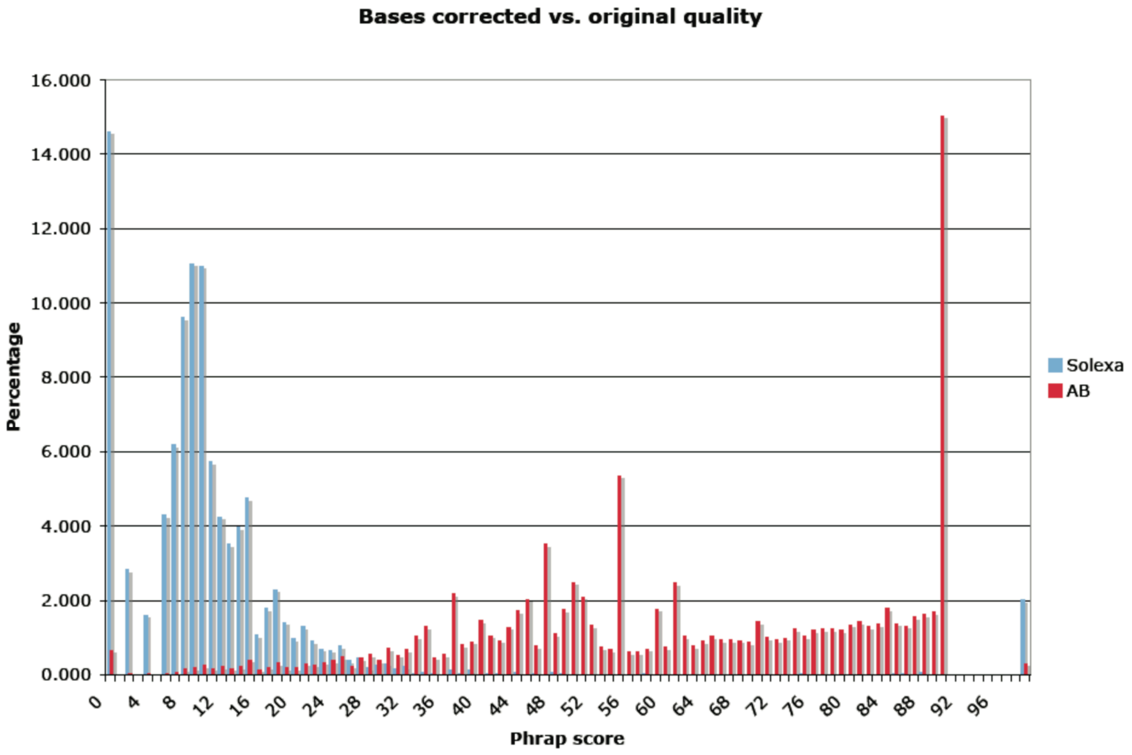

Figure S2 Sequence corrections. The phrap scores from the sequencing of the *A. gossypii* genome as reported previously (Dietrich et al. 2004) are shown in red. Shown in blue are the percentage of sequence corrections correlated to the phrap score of the individual base. More than 99% of all changes to the sequence, not including the telomeric additions, correspond to sequence positions of phrap score less than 30 in the original sequence.

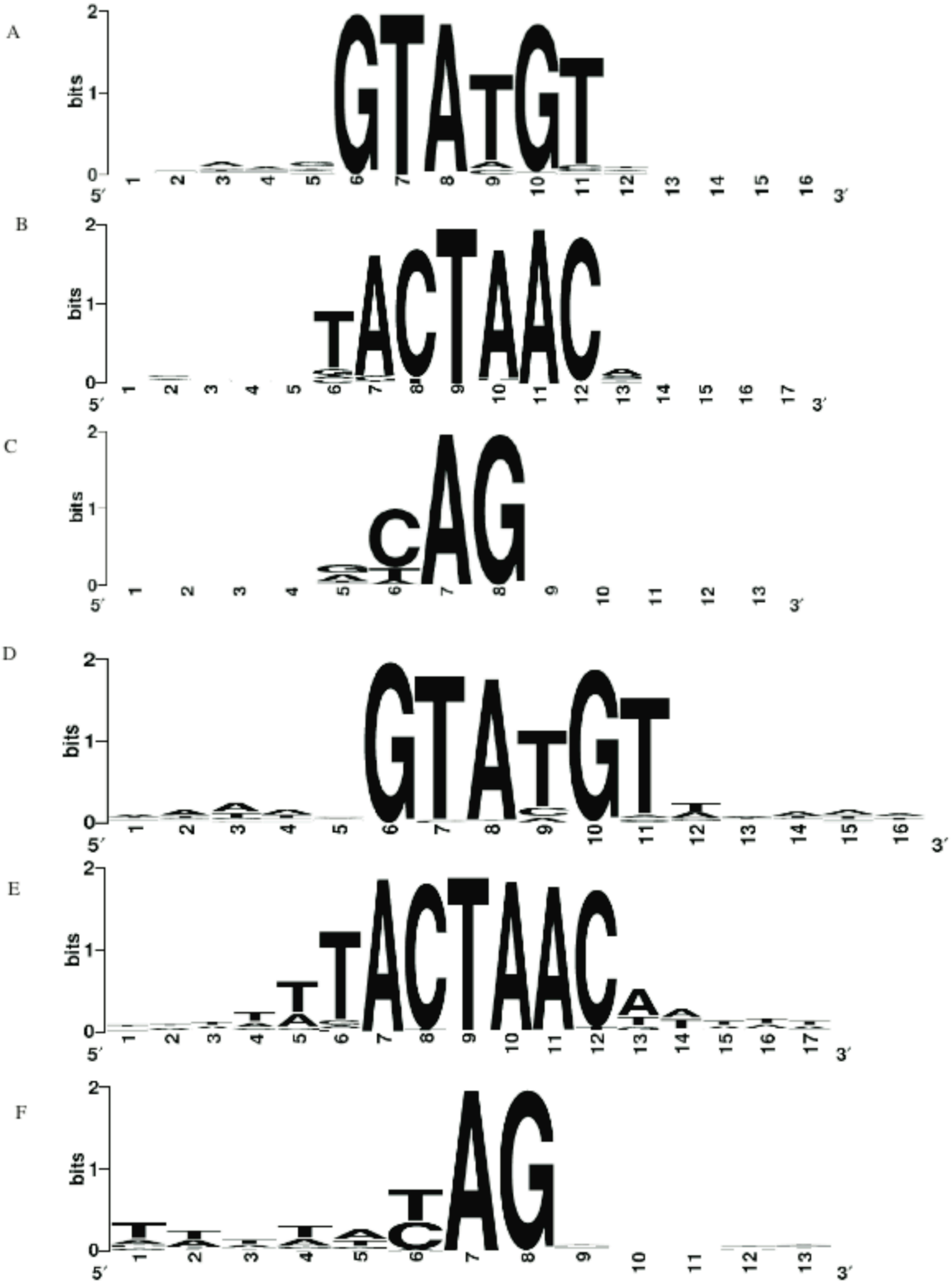

Figure S3 Intron Splice sites from *A. gossypii* and *S. cereivisiae.* Sequences were generated from the complete set of introns from protein coding genes and 5' UTR regions from both species. (A) 5' Spice site in *A. gossypii.* (B) Branch site in *A. gossypii.* (C) 3' splice site in *A. gossypii.* (D) 5' splice site in *S. cerevisiae.* (E) Branch site in *S. cerevisiae.* (F) 3' splice site in *S. cerevisiae.*

Table S1 SNPs and INDELs seen between strains ATCC10895 and insect isolate 1.

SNPs [1]												
nuclear:	A->G	G->A	C->T	T->C	A->C	A->T	G->C	G->T	C->A	C->G	T->A	T->G
Number:	2402	2456	2394	2364	721	646	646	768	780	748	659	753
Fraction:	0.16	0.16	0.16	0.15	0.05	0.04	0.04	0.05	0.05	0.05	0.04	0.05
SNPs												
Mito:	A->G	G->A	C->T	T->C	A->C	A->T	G->C	G->T	C->A	C->G	T->A	T->G
Number:	44	33	16	15	8	112	0	13	16	0	100	8
Fraction:	0.12	0.09	0.04	0.04	0.02	0.31	0	0.04	0.04	0	0.27	0.02
INDEL [2]												
nuclear:	1bp-D	2bp-D	3bp-D	4bp-D	>4bp-D	1bp-I	2bp-I	3bp-I	4bp-I	>4bp-I		
Number:	200	90	62	33	244	224	114	59	31	322		
INDEL												
Mito:	1bp-D	2bp-D	3bp-D	4bp-D	>4bp-D	1bp-I	2bp-I	3bp-I	4bp-I	>4bp-I		
Number:	25	11	10	7	34	32	17	11	8	28		

[1] The distribution of these 15702 substitution differences across the 9,211,222 bases of the Agleu2Δthr4Δ genome is an average of one SNP every 586 bases. There are too few SNP's in any one open reading frame to identify any genes with an unusual ratio of synonymous to non-synonymous changes with any statistical significance.

[2] INDELs. Number of insertion/deletion differences seen between strains Agleu2Δthr4Δ and insect isolate 1. 1bp-D means that strain Agleu2Δthr4Δ is missing one base relative to strain insect isolate 1. Of these 626 nuclear indels, only 39 are in the 81% of the genome that is protein coding regions, spans non-coding RNA genes, or centromeres, 19 of which are multiples of 3 bases in length, and two of which are nearby single base insertion/deletion pairs; the non-coding regions of the genome are thus significantly enhanced for INDELs.

Table S2 Changes to names of mating type and mating type associated *A. gossypii* genes.

A.g. ORF name	S.c. homolog	Alternate homolog	S.c. homolog common name	New name	Alternate gene
**Mating-type locus 1, right arm chr. VI**				MAT1	
AFR643W-**B**	YLR154C		RNH203	RNH203A	
AFR643W-A			(*K. lactis* MATa2)	MAT1a2	
AFR643C	YCR097W		HMRA1	MAT1a1	
AFR642C	YNL244C		SUI1	SUI1A	
AFR641W	YNL246W		VPS75	VPS75A	

**Mating-type locus 2, left telomere chr. IV**				MAT2	
ADL393W-A	YLR154C		RNH203	RNH203B	
ADL393W			(*K. lactis* MATa2)	MAT2a2	
ADL394C	YCR097W		HMRA1	MAT2a1	
ADL395C	YNL244C		SUI1	SUI1B	
ADL396W	YNL246W		VPS75	VPS75B	

**Mating-type locus 3, right telomere chr. V**				MAT3	
AER455C-A	YLR154C		RNH203	RNH203C	
AER455C			(*K. lactis* MATa2)	MAT3a2	
AER456W	YCR097W		HMRA1	MAT3a1	
AER457W	YNL244C		SUI1	SUI1C	
AER458C	YNL246W		VPS75	VPS75C	

**Mating-type locus 4, right telomere chr.VI**				MAT4	
AFR749C	YLR154C		RNH203	RNH203D	
AFR750C		YCR039C	(*K. lactis* MATa2)	MAT4a2	MAT4α2
AFR751W	YCR097W	YCR040W	HMRA1/MATalpha	MAT4a1	MAT4α1
AFR752W	YNL244C		SUI1	SUI1D	
AFR753C	YNL246W		VPS75	VPS75D	

Table S3 Names of repeated *A. gossypii* genes with a single *S. cerevisiae* homolog

#	A.g. ORF name	S.c.1° homolog	S.c.2° homolog	S.c.1° homolog common name	S.c.2° homolog common name	New Ashbya name [1]	
1	ACR282C	YAL018C	YOL047C/ YOL048C			LLP1a	Membrane proteins (likely at lipid particles)
2	ACR283W	YAL018C	YOL047C/ YOL048C			LLP1b	
3	ADL027W	YAL018C				LLP10	
4	ADR122C	YAL018C			RRT8	LLP11	
5	ADR403C	YAL051W	YOR363C	OAF1	PIP2	OAF1/ PIP2c	Oleate-activated transcription factors
6	ADR404C	YAL051W	YOR363C	OAF1	PIP2	OAF1/ PIP2b	
7	ADR405C	YAL051W	YOR363C	OAF1	PIP2	OAF1/ PIP2a	
8	AFR013C	YBL022C		PIM1		PIM1	Mitochodrial proteases
9	AFL121W	YBL022C		PIM1		PIM10	
10	AGR368W	YBL064C		PRX1		PRX1	Mitochondrial peroxiredoxins
11	ACL197W	YBL064C		PRX1		PRX10	
12	AER018C	YBR015C		MNN2		MNN2	alpha-1,2-mannosyltransferases
13	AEEL082W	YBR015C		MNN2		MNN20	
14	AAL128C	YBR072W		HSP26		HSP26E	Heat shock proteins (chaperon activity)
15	ADL397C	YBR072W		HSP26		HSP26B	
16	AER459W	YBR072W		HSP26		HSP26C	
17	AFR437W	YBR072W		HSP26		HSP26F	
18	AFR633W	YBR072W		HSP26		HSP26A	
19	AFR754W-A	YBR072W		HSP26		HSP26D	
17	ABR185W	YCL057W		PRD1		PRD1	Endopeptidases
18	ABR405C	YCL057W		PRD1		PRD10b	
19	AGR406C	YCL057W		PRD1		DRD10a	
20	ADR197W	YCR098C		GIT1		GIT10	Glycerol-P-inosital transporters (similar to GIT1)
21	AER386W	YCR098C		GIT1		GIT11	
22	ABL189W	YDL237W		AIM6		AIM6a	Required for respiratory growth
23	ABL188W	YDL237W		AIM6		AIM6b	
24	ABR125C	YDR011W	YNR070W	SNQ2		SNQ20	ABC transporters (similar to SNQ2)
25	AFR326W	YDR011W	YNR070W	SNQ2		SNQ21	
26	AGR038C	YDR046C	YBR068C	BAP3	BAP2	BAP2/ BAP3b	Branched amino acid permeases
27	AGR039C	YDR046C	YBR068C	BAP3	BAP2	BAP2/ BAP3a	
28	AGR188W	YDR227W		SIR4		SIR4a	Silent information regulator
29	AGR189W	YDR227W		SIR4		SIR4b	

30	ACR086C	YDR270W		CCC2		CCC2	Copper transporting ATPases
31	AGL041C	YDR270W		CCC2		CCC20	
32	AFR698C	YDR508C	YCL025C	GNP1	AGP1	AGP1	Low affinity aminoacis permeases
33	ADL272W	YDR508C	YCL025C	GNP1	AGP1	AGP10	
34	AFR366W	YEL046C		GLY1		GLY1	Glycin biosynthsis (threonine aldolases)
35	AGR200W	YEL046C		GLY1		GLY10	
36	AGR129C	YER145C		FTR1		FTR1	High-affinity iron transporters
37	AEL294C	YER145C		FTR1		FTR10	
38	ABR228C	YFL041W		FET5		FET5	Multicopper oxidases (role in iron transport)
39	ADR239W	YFL041W		FET5		FET50	
40	AAR153C	YGL055W		OLE1		OLE1	Fatty acid desaturases
41	AAL078W	YGL055W		OLE1		OLE10	
42	AFR548C	YGL202W		ARO8		ARO8	Aromatic aminotransferases
43	AGR167W	YGL202W		ARO8		ARO81	
44	AFR262C	YGL246C		RAI1		RAI1b	Decapping of mRNAs
45	AFR263C	YGL246C		RAI1		RAI1a	
46	AGR021C	YGR012W				MCY1	Putative mitochondrial cystein synthases
47	AEL161W	YGR012W				MCY10	
48	ACL181C	YGR032W	YLR342W	GSC2 (FKS2)	FKS1	FKS1/ FKS2	beta-1,3-glucan synthases
49	AAR035W	YLR342W	YGR032W	FKS1	GSC2 (FKS2)	FKS20	
50	ADR330W	YGR260W		TNA1		TNA1	Nicotinic acid transporters
51	AGL026W	YGR260W		TNA1		TNA10	
52	ABR009W	YHL003C	YKL008C	LAG1	LAC1	LAG1/ LAC1	Ceramid synthesis
53	ADL206W	YHL003C		LAG1		LAG10	
54	AAL036C	YHR032W		ERC1		ERC1	Multi-drug exporters
55	AER234W	YHR032W		ERC1		ERC10	
56	AFL092C	YHR211W		FLO5		FLO5a	Cell wall flocculins
57	AFL095W	YHR211W		FLO5		FLO5b	
58	AFL020C	YIL014W		MNT3		MNT3	alpha-1,3-mannosyltransferases
59	AFL235W	YIL014W		MNT3		MNT30	
60	AAR183C	YIL014W		MNT3		MNT31	
61	ABR245C	YIL014W		MNT3		MNT32	
62	AEL345W	YIL014W		MNT3		MNT33	
63	AGL364C	YIL159W		BNR1		BNR2	Formins (BNI1-related)
64	AFR301C	YIL159W		BNR1		BNR1	
65	ACL203C	YIL166C	YOL162/ YOL163W			DAL50	Members of the DAL5 family of facilitators
66	AER444W	YIL166C	YOL162/ YOL163W			DAL51	
67	AFR229C	YIL166C	YOL162/			DAL52	

			YOL163W				
68	AGR235W	YIL166C	YOL162/ YOL163W			DAL53	
69	ABR246W	YIR035C	YIR036C		IRC24	NRE10a	Putative novel reductases
70	ABR247W	YIR035C	YIR036C		IRC24	NRE10b	
71	ABR248W	YIR035C	YIR036C		IRC24	NRE10c	
72	ABR249W	YIR035C	YIR036C		IRC24	NRE10d	
73	ACR171C	YIR035C	YIR036C		IRC24	NRE1	
74	AAL179W	YJL078C	YKR013W	PRY3	PRY2	PRY3	Acetylated sterol transporters (cell wall)
75	AAL178W	YJL079C	YKR013W	PRY1	PRY2	PRY1	
76	AEL132W	YJL172W		CPS1		CPS1	Vacuolar peptidases
77	AGL326W	YJL172W		CPS1		CPS10a	
78	AGL325W	YJL172W		CPS1		CPS10b	
79	AFR644C	YJL204C		RCY1		RCY1	Recycling of plasma menbrane
80	AFR748W	YJL204C		RCY1		RCY10	
81	AER445C	YJR076C		CDC11		CDC11a	Duplicated septins
82	AFR436C	YJR076C		CDC11		CDC11b	
83	AGL316W	YJR099W		YUH1		YUH1a	
84	AGL314C	YJR099W		YUH1		YUH1b	
85	ABR159C	YJR107W				PLI1	Putative lipases
86	ACL114W	YJR107W				PIL10	
87	AER452C	YJR107W				PIL11c	
88	AER453C	YJR107W				PIL11b	
89	AER454C	YJR107W				PIL11a	
90	ABR025C	YKL096W		CWP1		CWP1d	Structural cell wall proteins
91	ABR026C	YKL096W		CWP1		CWP1c	
92	ABR027C	YKL096W		CWP1		CWP1b	
93	ABR028C	YKL096W		CWP1		CWP1a	
94	ACR272W	YKL096W		CWP1		CWP10a	
95	ACR073W	YKL096W		CWP1		CWP10b	
96	ADL398C	YKL096W		CWP1		CWP11	
97	AFR756W	YKL096W		CWP1		CWP12	
98	AAR192C	YKL217W		JEN1		JEN10	Monocarboxylate symsporters (similar to JEN1)
99	ABL210C	YKL217W		JEN1		JEN11	
100	AFR333W	YKL217W		JEN1		JEN12	
101	ADR081C	YLR215C		CDC123		CDC123b	Nutritional control of the cell cycle
102	ADR082C	YLR215C		CDC123		CDC123a	
103	AFR530W	YMR238W		DFG5		DFG5	Mannosidases (linkage of GPI proteins to cell wall)
104	ACL202W	YMR238W		DFG5		DFG50a	
105	ACL201W	YMR238W		DFG5		DFG50b	
106	ACL200W	YMR238W		DFG5		DFG50c	

107	AGL213W	YMR252C				MLO1	putative mitrochondrial located proteins
108	AFR125C	YMR252C				MLO10	
109	ABL117C	YMR284W		YKU70		YKU70	Subunit of telomeric KU complex (DSB repair)
110	AFR443C	YMR284W		YKU70		YKU71	
111	AGL351W	YMR307W		GAS1		GAS1a	beta-1,3-transglycosidases
112	AGL352W	YMR307W		GAS1		GAS1b	
113	AFL229W	YNL104C	YOR018W	LEU4	LEU9	LEU4	First step in leucine biosynthesis
114	ADL015C	YNL104C		LEU4		LEU40	
115	AFR682C	YNL277W		MET2		MET2	First step in methionine biosynthesis
116	AEL098W	YNL277W		MET2		MET20	
117	ADR336C	YNR055C		HOL1		HOL1b	Members of the antiporter DHA1 family
118	ADR337C	YNR055C		HOL1		HOL1a	
119	AGL069C	YNR055C		HOL1		HOL10	
120	ADL156C	YOL119C		MCH4		MCH4b	Monocarboxylate transporters
121	ADL155C	YOL119C		MCH4		MCH4a	
122	ADL095W	YOL119C		MCH4		MCH40	
123	ACR098C	YPL129W	YOR213C	TAF14 (ANC1)	SAS5	TAF14/ SAS5a	Complex subunits with YEATS domain
124	ACR099C	YPL129W	YOR213C	TAF14 (ANC1)	SAS5	TAF14/ SAS5b	
125	ACR143W	YPL154C		PEP4		PEP4a	Vacuolar aspartyl proteases
126	ACR144W	YPL154C		PEP4		PEP4b	
127	ABL123C	YPL154C		PEP4		PEP40	
128	AGR407C	YPL154C		PEP4		PEP41	
129	ABL125W	YPL273W		SAM4		SAM40	S-Adenosyl-Methionine metabolism
130	AFR410W	YPL273W		SAM4		SAM41	
131	ABR182W	YPR165W		RHO1		RHO1a	Rho proteins (regulator of beta-1,3-glucan synthases)
132	ABR183W	YPR165W		RHO1		RHO1b	
133	ADL399C	YPR194C		OPT2		OPT20	Putative oligo peptide transporters
134	AFR757W	YPR194C		OPT2		OPT21	
135	ACL205C	YPR194C		OPT2		OPT22	
136	AGL027W	YPR194C		OPT2		OPT23	

[1] Rules for naming of repeated *Ashbya* genes

A. The syntenic copy keeps the *S. cerevisiae* name.

B. The non-syntenic copy keeps the three letters and adds to the number 0, 1, 2, e.g. MNN2 or LEU4 for the syntenic copies and MNN20 or LEU40 for the non-synthenic copies. Or MNT3 for the syntenic copy and MNT30 to 33 for the four non-syntenic copies that are in this case mainly at different telomeres.

C. In the absence of syntenic copies the original *S. cerevisiae* name is not given however the three letter code remains with added higher numbers, e.g. JEN10, JEN11 and JEN12 for the three non-syntenic homologs of JEN1, or GIT10 and 11 for the two non-syntenic homologs of GIT1.
D. In case of tandem gene amplifications at syntenic loci small letters (a, b, c etc) were added following the convention, that a is the leftmost copy when the direction of transcription is drawn from left to right.
E. For the six HSP26 copies (all non-syntenic) the well know name was kept (exception to the rule) and capital letters A to F were added.
