# Supplementary material for: Genomes of Ashbya Fungi Isolated from Insects Reveal Four Mating-Type Loci, Numerous Translocations, Lack of Transposons, and Distinct Gene Duplications
Source: G3 (Bethesda). 2013 Aug 1;3(8):1225–39. doi: 10.1534/g3.112.002881 (PMC3737163; doi:10.1534/g3.112.002881)
Supplement: Supporting Information [file supp_g3.112.002881_FigureS1.pdf]

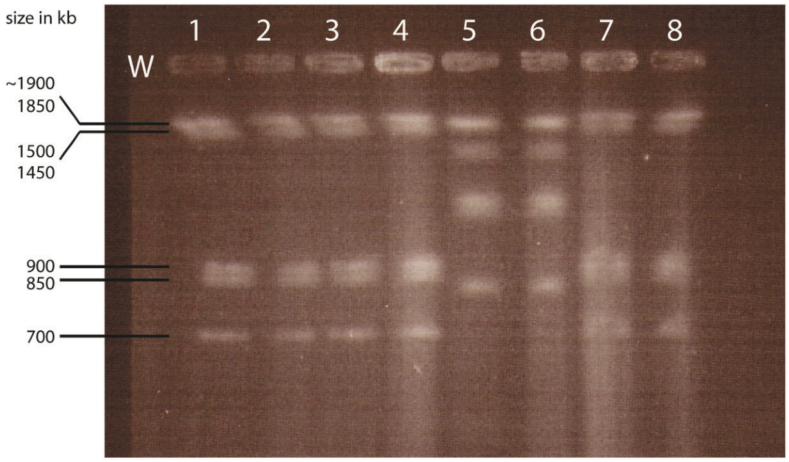

Figure S1 Pulsed field gel of *A. gossypii* strain ATCC10895 (lanes 7,8) and of insect isolate 1 (lanes 1,2), *A. aceri* (lanes 5, 6), and an *A. gossypii* isolate from a milk weed bug living on oleander in Vera Beach Florida (lanes 3,4) Chromosomal size heterogeneity is apparent between these species. W indicates the position of the loading wells. Sizes based on sizes of the *A. gossypii* strain ATCC10895 chromosomes.
