# Supplementary material for: Genomes of Ashbya Fungi Isolated from Insects Reveal Four Mating-Type Loci, Numerous Translocations, Lack of Transposons, and Distinct Gene Duplications
Source: G3 (Bethesda). 2013 Aug 1;3(8):1225–39. doi: 10.1534/g3.112.002881 (PMC3737163; doi:10.1534/g3.112.002881)
Supplement: Supporting Information [file supp_g3.112.002881_FigureS2.pdf]

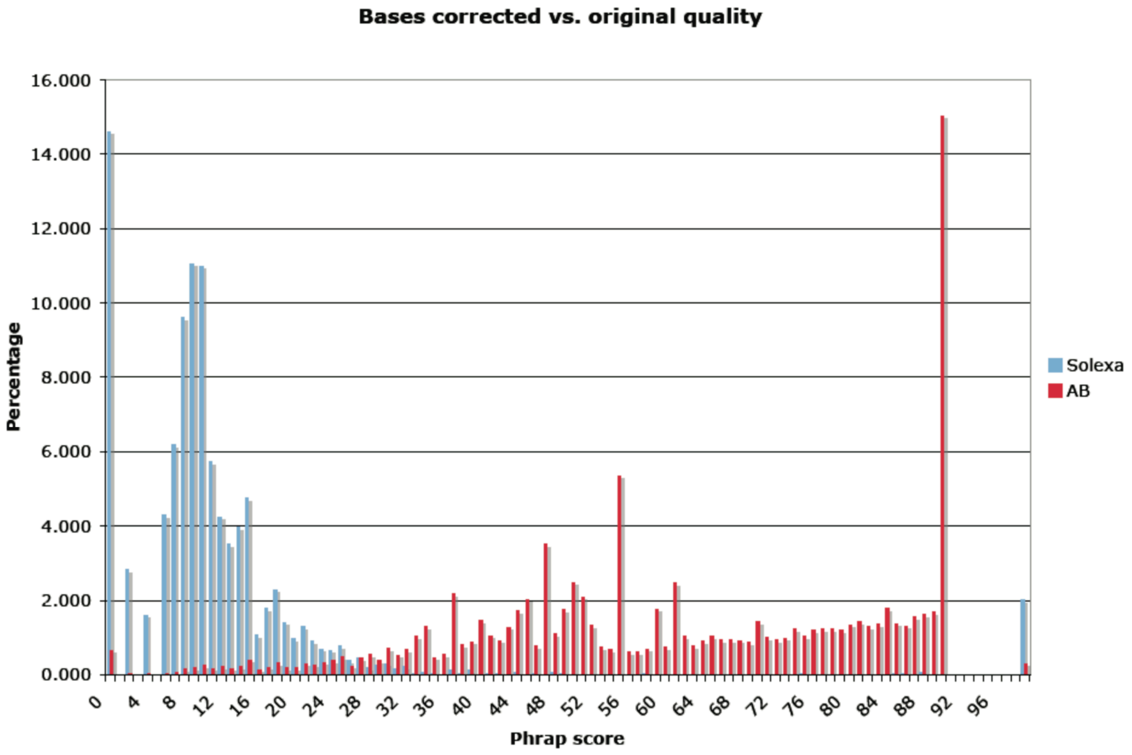

Figure S2 Sequence corrections. The phrap scores from the sequencing of the *A. gossypii* genome as reported previously (Dietrich et al. 2004) are shown in red. Shown in blue are the percentage of sequence corrections correlated to the phrap score of the individual base. More than 99% of all changes to the sequence, not including the telomeric additions, correspond to sequence positions of phrap score less than 30 in the original sequence.
