# Supplementary material for: Genomes of Ashbya Fungi Isolated from Insects Reveal Four Mating-Type Loci, Numerous Translocations, Lack of Transposons, and Distinct Gene Duplications
Source: G3 (Bethesda). 2013 Aug 1;3(8):1225–39. doi: 10.1534/g3.112.002881 (PMC3737163; doi:10.1534/g3.112.002881)
Supplement: Supporting Information [file supp_g3.112.002881_FigureS3.pdf]

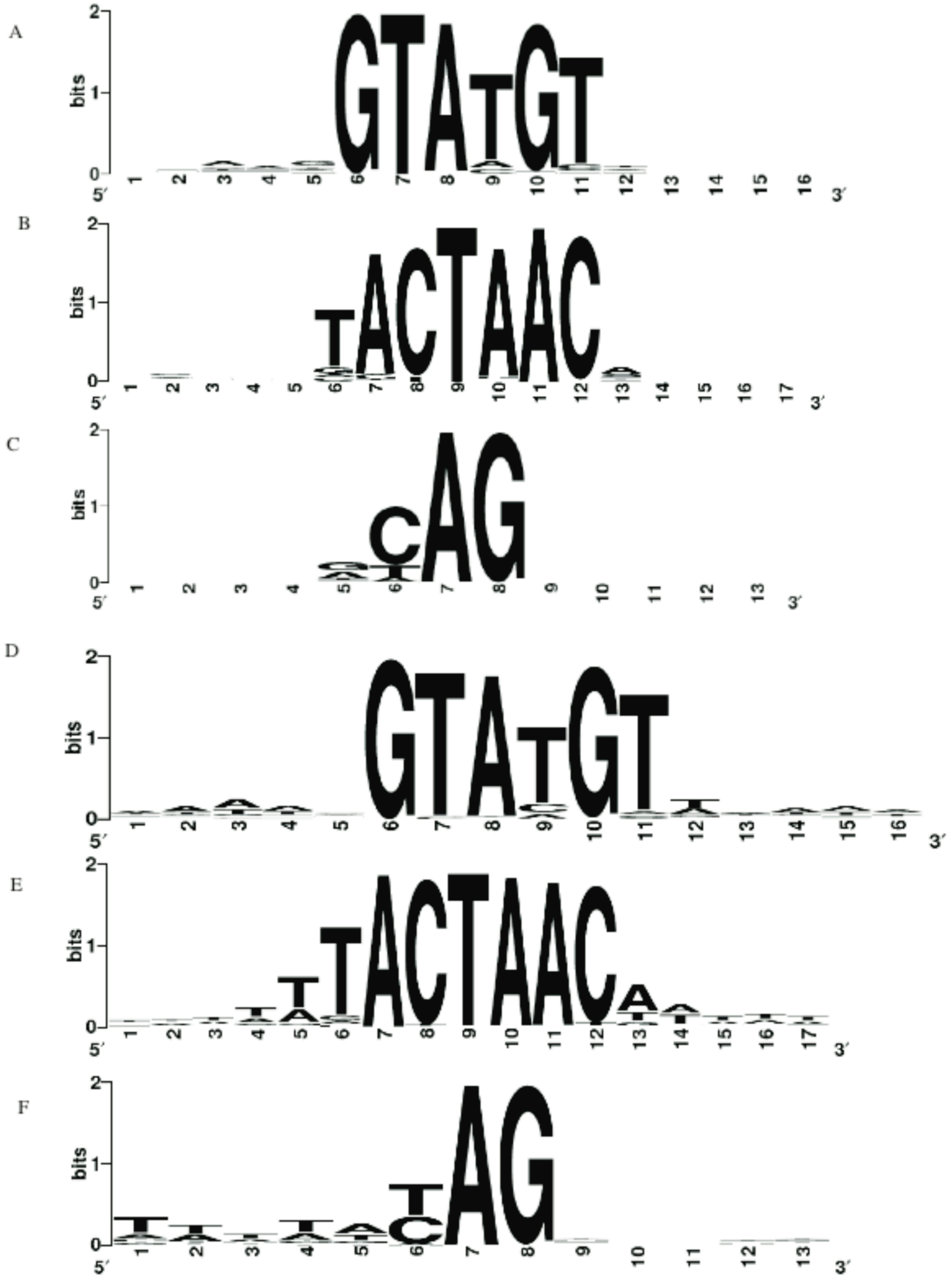

Figure S3 Intron Splice sites from *A. gossypii* and *S. cereivisiae.* Sequences were generated from the complete set of introns from protein coding genes and 5' UTR regions from both species. (A) 5' Spice site in *A. gossypii.* (B) Branch site in *A. gossypii.* (C) 3' splice site in *A. gossypii.* (D) 5' splice site in *S. cerevisiae.* (E) Branch site in *S. cerevisiae.* (F) 3' splice site in *S. cerevisiae.*
