# Supplementary material for: Genomes of Ashbya Fungi Isolated from Insects Reveal Four Mating-Type Loci, Numerous Translocations, Lack of Transposons, and Distinct Gene Duplications
Source: G3 (Bethesda). 2013 Aug 1;3(8):1225–39. doi: 10.1534/g3.112.002881 (PMC3737163; doi:10.1534/g3.112.002881)
Supplement: Supporting Information [file supp_g3.112.002881_TableS1.pdf]

Table S1 SNPs and INDELs seen between strains ATCC10895 and insect isolate 1.

SNPs [1]												
nuclear:	A->G	G->A	C->T	T->C	A->C	A->T	G->C	G->T	C->A	C->G	T->A	T->G
Number:	2402	2456	2394	2364	721	646	646	768	780	748	659	753
Fraction:	0.16	0.16	0.16	0.15	0.05	0.04	0.04	0.05	0.05	0.05	0.04	0.05
SNPs												
Mito:	A->G	G->A	C->T	T->C	A->C	A->T	G->C	G->T	C->A	C->G	T->A	T->G
Number:	44	33	16	15	8	112	0	13	16	0	100	8
Fraction:	0.12	0.09	0.04	0.04	0.02	0.31	0	0.04	0.04	0	0.27	0.02

INDEL [2]										
nuclear:	1bp-D	2bp-D	3bp-D	4bp-D	>4bp-D	1bp-I	2bp-I	3bp-I	4bp-I	>4bp-I
Number:	200	90	62	33	244	224	114	59	31	322
INDEL										
Mito:	1bp-D	2bp-D	3bp-D	4bp-D	>4bp-D	1bp-I	2bp-I	3bp-I	4bp-I	>4bp-I
Number:	25	11	10	7	34	32	17	11	8	28

[1] The distribution of these 15702 substitution differences across the 9,211,222 bases of the Agleu2Δthr4Δ genome is an average of one SNP every 586 bases. There are too few SNP's in any one open reading frame to identify any genes with an unusual ratio of synonymous to non-synonymous changes with any statistical significance.

[2] INDELs. Number of insertion/deletion differences seen between strains Agleu2Δthr4Δ and insect isolate 1. 1bp-D means that strain Agleu2Δthr4Δ is missing one base relative to strain insect isolate 1. Of these 626 nuclear indels, only 39 are in the 81% of the genome that is protein coding regions, spans non-coding RNA genes, or centromeres, 19 of which are multiples of 3 bases in length, and two of which are nearby single base insertion/deletion pairs; the non-coding regions of the genome are thus significantly enhanced for INDELs.
