# Supplementary material for: Genomes of Ashbya Fungi Isolated from Insects Reveal Four Mating-Type Loci, Numerous Translocations, Lack of Transposons, and Distinct Gene Duplications
Source: G3 (Bethesda). 2013 Aug 1;3(8):1225–39. doi: 10.1534/g3.112.002881 (PMC3737163; doi:10.1534/g3.112.002881)
Supplement: Supporting Information [file supp_g3.112.002881_TableS2.pdf]

Table S2 Changes to names of mating type and mating type associated *A. gossypii* genes.

A.g. ORF name	S.c. homolog	Alternate homolog	S.c. homolog common name	New name	Alternate gene
**Mating-type locus 1, right arm chr. VI**				MAT1	
AFR643W-**B**	YLR154C		RNH203	RNH203A	
AFR643W-A			(*K. lactis* MATa2)	MAT1a2	
AFR643C	YCR097W		HMRA1	MAT1a1	
AFR642C	YNL244C		SUI1	SUI1A	
AFR641W	YNL246W		VPS75	VPS75A	
**Mating-type locus 2, left telomere chr. IV**				MAT2	
ADL393W-A	YLR154C		RNH203	RNH203B	
ADL393W			(*K. lactis* MATa2)	MAT2a2	
ADL394C	YCR097W		HMRA1	MAT2a1	
ADL395C	YNL244C		SUI1	SUI1B	
ADL396W	YNL246W		VPS75	VPS75B	
**Mating-type locus 3, right telomere chr. V**				MAT3	
AER455C-A	YLR154C		RNH203	RNH203C	
AER455C			(*K. lactis* MATa2)	MAT3a2	
AER456W	YCR097W		HMRA1	MAT3a1	
AER457W	YNL244C		SUI1	SUI1C	
AER458C	YNL246W		VPS75	VPS75C	
**Mating-type locus 4, right telomere chr.VI**				MAT4	
AFR749C	YLR154C		RNH203	RNH203D	
AFR750C		YCR039C	(*K. lactis* MATa2)	MAT4a2	MAT4α2
AFR751W	YCR097W	YCR040W	HMRA1/MATalpha	MAT4a1	MAT4α1
AFR752W	YNL244C		SUI1	SUI1D	
AFR753C	YNL246W		VPS75	VPS75D	
