# Supplementary material for: Genomes of Ashbya Fungi Isolated from Insects Reveal Four Mating-Type Loci, Numerous Translocations, Lack of Transposons, and Distinct Gene Duplications
Source: G3 (Bethesda). 2013 Aug 1;3(8):1225–39. doi: 10.1534/g3.112.002881 (PMC3737163; doi:10.1534/g3.112.002881)
Supplement: Supporting Information [file supp_g3.112.002881_TableS3.pdf]

Table S3 Names of repeated *A. gossypii* genes with a single *S. cerevisiae* homolog

#	A.g. ORF name	S.c.1° homolog	S.c.2° homolog	S.c.1° homolog common name	S.c.2° homolog common name	New Ashbya name [1]	
1	ACR282C	YAL018C	YOL047C/ YOL048C			LLP1a	Membrane proteins (likely at lipid particles)
2	ACR283W	YAL018C	YOL047C/ YOL048C			LLP1b	
3	ADL027W	YAL018C				LLP10	
4	ADR122C	YAL018C			RRT8	LLP11	
5	ADR403C	YAL051W	YOR363C	OAF1	PIP2	OAF1/ PIP2c	Oleate-activated transcription factors
6	ADR404C	YAL051W	YOR363C	OAF1	PIP2	OAF1/ PIP2b	
7	ADR405C	YAL051W	YOR363C	OAF1	PIP2	OAF1/ PIP2a	
8	AFR013C	YBL022C		PIM1		PIM1	Mitochondrial proteases
9	AFL121W	YBL022C		PIM1		PIM10	
10	AGR368W	YBL064C		PRX1		PRX1	Mitochondrial peroxiredoxins
11	ACL197W	YBL064C		PRX1		PRX10	
12	AER018C	YBR015C		MNN2		MNN2	alpha-1,2-mannosyltransferases
13	AEEL082W	YBR015C		MNN2		MNN20	
14	AAL128C	YBR072W		HSP26		HSP26E	Heat shock proteins (chaperon activity)
15	ADL397C	YBR072W		HSP26		HSP26B	
16	AER459W	YBR072W		HSP26		HSP26C	
17	AFR437W	YBR072W		HSP26		HSP26F	
18	AFR633W	YBR072W		HSP26		HSP26A	
19	AFR754W-A	YBR072W		HSP26		HSP26D	
20	ABR185W	YCL057W		PRD1		PRD1	Endopeptidases
21	AGR405C	YCL057W		PRD1		PRD10b	
22	AGR406C	YCL057W		PRD1		PRD10a	
23	ADR197W	YCR098C		GIT1		GIT10	Glycerophosphoinositol transporters (similar to GIT1)
24	AER386W	YCR098C		GIT1		GIT11	
25	ABL189W	YDL237W		AIM6		AIM6a	Required for respiratory growth
26	ABL188W	YDL237W		AIM6		AIM6b	
27	ABR125C	YDR011W	YNR070W	SNQ2		SNQ20	ABC transporters (similar to SNQ2)
28	AFR326W	YDR011W	YNR070W	SNQ2		SNQ21	
29	AGR038C	YDR046C	YBR068C	BAP3	BAP2	BAP2/ BAP3b	Branched amino acid permeases
30	AGR039C	YDR046C	YBR068C	BAP3	BAP2	BAP2/ BAP3a	
31	AGR188W	YDR227W		SIR4		SIR4a	Silent information regulator
32	AGR189W	YDR227W		SIR4		SIR4b	

33	ACR086C	YDR270W		CCC2		CCC2	Copper transporting ATPases
34	AGL041C	YDR270W		CCC2		CCC20	
35	AFR698C	YDR508C	YCL025C	GNP1	AGP1	AGP1	Low affinity amino acid permeases
36	ADL272W	YDR508C	YCL025C	GNP1	AGP1	AGP10	
37	AFR366W	YEL046C		GLY1		GLY1	Glycine biosynthesis (threonine aldolases)
38	AGR200W	YEL046C		GLY1		GLY10	
39	AGR129C	YER145C		FTR1		FTR1	High-affinity iron transporters
40	AEL294C	YER145C		FTR1		FTR10	
41	ABR228C	YFL041W		FET5		FET5	Multicopper oxidases (role in iron transport)
42	ADR239W	YFL041W		FET5		FET50	
43	AAR153C	YGL055W		OLE1		OLE1	Fatty acid desaturases
44	AAL078W	YGL055W		OLE1		OLE10	
45	AFR548C	YGL202W		ARO8		ARO8	Aromatic amino transferases
46	AGR167W	YGL202W		ARO8		ARO81	
47	AFR262C	YGL246C		RAI1		RAI1b	Decapping of mRNAs
48	AFR263C	YGL246C		RAI1		RAI1a	
49	AGR021C	YGR012W				MCY1	Putative mitochondrial cystein synthases
50	AEL161W	YGR012W				MCY10	
51	ACL181C	YGR032W	YLR342W	GSC2 (FKS2)	FKS1 (GSC1)	FKS1/ FKS2	beta-1,3-glucan synthases
52	AAR035W	YLR342W	YGR032W	FKS1 (GSC1)	GSC2 (FKS2)	FKS10	
53	ADR330W	YGR260W		TNA1		TNA1	Nicotinic acid transporters
54	AGL026W	YGR260W		TNA1		TNA10	
55	ABR009W	YHL003C	YKL008C	LAG1	LAC1	LAG1/ LAC1	Ceramide synthesis
56	ADL206W	YHL003C		LAG1		LAG10	
57	AAL036C	YHR032W		ERC1		ERC1	Multi-drug exporters
58	AER234W	YHR032W		ERC1		ERC10	
59	AFL092C	YHR211W		FLO5		FLO5a	Cell wall flocculins
60	AFL095W	YHR211W		FLO5		FLO5b	
61	AFL020C	YIL014W		MNT3		MNT3	alpha-1,3-mannosyltransferases
62	AFL235W	YIL014W		MNT3		MNT30	
63	AAR183C	YIL014W		MNT3		MNT31	
64	ABR245C	YIL014W		MNT3		MNT32	
65	AEL345W	YIL014W		MNT3		MNT33	
66	AGL364C	YIL159W		BNR1		BNR2	Formins (BNI1-related)
67	AFR301C	YIL159W		BNR1		BNR1	
68	ACL203C	YIL166C	YOL162/ YOL163W			DAL50	Members of the DAL5 family of facilitators
69	AER444W	YIL166C	YOL162/ YOL163W			DAL51	
70	AFR229C	YIL166C	YOL162/			DAL52	

			YOL163W				
71	AGR235W	YIL166C	YOL162/ YOL163W			DAL53	
72	ABR246W	YIR035C	YIR036C		IRC24	NRE10a	Putative novel reductases
73	ABR247W	YIR035C	YIR036C		IRC24	NRE10b	
74	ABR248W	YIR035C	YIR036C		IRC24	NRE10c	
75	ABR249W	YIR035C	YIR036C		IRC24	NRE10d	
76	ACR171C	YIR035C	YIR036C		IRC24	NRE1	
77	AAL179W	YJL078C	YKR013W	PRY3	PRY2	PRY3	Acetylated sterol transporters (cell wall)
78	AAL178W	YJL079C	YKR013W	PRY1	PRY2	PRY1	
79	AEL132W	YJL172W		CPS1		CPS1	Vacuolar peptidases
80	AGL326W	YJL172W		CPS1		CPS10a	
81	AGL325W	YJL172W		CPS1		CPS10b	
82	AFR644C	YJL204C		RCY1		RCY1	Recycling of plasma membrane
83	AFR748W	YJL204C		RCY1		RCY10	
84	AER445C	YJR076C		CDC11		CDC11a	Duplicated septins
85	AFR436C	YJR076C		CDC11		CDC11b	
86	AGL316W	YJR099W		YUH1		YUH1a	
87	AGL314C	YJR099W		YUH1		YUH1b	
88	ABR159C	YJR107W				PLI1	Putative lipases
89	ACL114W	YJR107W				PLI10	
90	AER452C	YJR107W				PLI11c	
91	AER453C	YJR107W				PLI11b	
92	AER454C	YJR107W				PLI11a	
93	ABR025C	YKL096W		CWP1		CWP1d	Structural cell wall proteins
94	ABR026C	YKL096W		CWP1		CWP1c	
95	ABR027C	YKL096W		CWP1		CWP1b	
96	ABR028C	YKL096W		CWP1		CWP1a	
97	ACR272W	YKL096W		CWP1		CWP10a	
98	ACR273W	YKL096W		CWP1		CWP10b	
99	ADL398C	YKL096W		CWP1		CWP11	
100	AFR756W	YKL096W		CWP1		CWP12	
101	AAR192C	YKL217W		JEN1		JEN10	Monocarboxylate symporters (similar to JEN1)
102	ABL210C	YKL217W		JEN1		JEN11	
103	AFR333W	YKL217W		JEN1		JEN12	
104	ADR081C	YLR215C		CDC123		CDC123b	Nutritional control of the cell cycle
105	ADR082C	YLR215C		CDC123		CDC123a	
106	AFR530W	YMR238W		DFG5		DFG5	Mannosidases (linkage of GPI proteins to cell wall)
107	ACL202W	YMR238W		DFG5		DFG50a	
108	ACL201W	YMR238W		DFG5		DFG50b	
109	ACL200W	YMR238W		DFG5		DFG50c	

110	AGL213W	YMR252C				MLO1	putative mitochondrial located proteins
111	AFR125C	YMR252C				MLO10	
112	ABL117C	YMR284W		YKU70		YKU70	Subunit of telomeric KU complex (DSB repair)
113	AFR443C	YMR284W		YKU70		YKU71	
114	AGL351W	YMR307W		GAS1		GAS1a	beta-1,3-transglycosidases
115	AGL352W	YMR307W		GAS1		GAS1b	
116	AFL229W	YNL104C	YOR018W	LEU4	LEU9	LEU4	First step in leucine biosynthesis
117	ADL015C	YNL104C		LEU4		LEU40	
118	AFR682C	YNL277W		MET2		MET2	First step in methionine biosynthesis
119	AEL098W	YNL277W		MET2		MET20	
120	ADR336C	YNR055C		HOL1		HOL1b	Members of the antiporter DHA1 family
121	ADR337C	YNR055C		HOL1		HOL1a	
122	AGL069C	YNR055C		HOL1		HOL10	
123	ADL156C	YOL119C		MCH4		MCH4b	Monocarboxylate transporters
124	ADL155C	YOL119C		MCH4		MCH4a	
125	ADL095W	YOL119C		MCH4		MCH40	
126	ACR098C	YPL129W	YOR213C	TAF14 (ANC1)	SAS5	TAF14/ SAS5a	Complex subunits with YEATS domain
127	ACR099C	YPL129W	YOR213C	TAF14 (ANC1)	SAS5	TAF14/ SAS5b	
128	ACR143W	YPL154C		PEP4		PEP4a	Vacuolar aspartyl proteases
129	ACR144W	YPL154C		PEP4		PEP4b	
130	ABL123C	YPL154C		PEP4		PEP40	
131	AGR407C	YPL154C		PEP4		PEP41	
132	ABL125W	YPL273W		SAM4		SAM40	S-Adenosyl-Methionine metabolism
133	AFR410W	YPL273W		SAM4		SAM41	
134	ABR182W	YPR165W		RHO1		RHO1a	Rho proteins (regulator of beta-1,3-glucan synthases)
135	ABR183W	YPR165W		RHO1		RHO1b	
136	ADL399C	YPR194C		OPT2		OPT20	Putative oligo peptide transporters
137	AFR757W	YPR194C		OPT2		OPT21	
138	ACL205C	YPR194C		OPT2		OPT22	
139	AGL027W	YPR194C		OPT2		OPT23	

[1] Rules for naming of repeated *Ashbya* genes

A. The syntenic copy keeps the *S. cerevisiae* name.

B. The non-syntenic copy keeps the three letters and adds to the number 0, 1, 2, e.g. MNN2 or LEU4 for the syntenic copies and MNN20 or LEU40 for the non-synthenic copies. Or MNT3 for the syntenic copy and MNT30 to 33 for the four non-syntenic copies that are in this case mainly at different telomeres.

C. In the absence of syntenic copies the original *S. cerevisiae* name is not given however the three letter code remains

with added higher numbers, e.g. JEN10, JEN11 and JEN12 for the three non-syntenic homologs of JEN1, or GIT10 and 11 for the two non-syntenic homologs of GIT1.
D. In case of tandem gene amplifications at syntenic loci small letters (a, b, c etc) were added following the convention that “a” is the leftmost copy when the direction of transcription is drawn from left to right.
E. For the six HSP26 copies (all non-syntenic) the well know name was kept (exception to the rule) and capital letters A to F were added.
.
